# Layer-by-layer epitaxy of multi-layer MoS_2_ wafers

**DOI:** 10.1093/nsr/nwac077

**Published:** 2022-04-21

**Authors:** Qinqin Wang, Jian Tang, Xiaomei Li, Jinpeng Tian, Jing Liang, Na Li, Depeng Ji, Lede Xian, Yutuo Guo, Lu Li, Qinghua Zhang, Yanbang Chu, Zheng Wei, Yanchong Zhao, Luojun Du, Hua Yu, Xuedong Bai, Lin Gu, Kaihui Liu, Wei Yang, Rong Yang, Dongxia Shi, Guangyu Zhang

**Affiliations:** Beijing National Laboratory for Condensed Matter Physics and Institute of Physics, Chinese Academy of Sciences, Beijing 100190, China; School of Physical Sciences, University of Chinese Academy of Sciences, Beijing 100190, China; Beijing National Laboratory for Condensed Matter Physics and Institute of Physics, Chinese Academy of Sciences, Beijing 100190, China; School of Physical Sciences, University of Chinese Academy of Sciences, Beijing 100190, China; Beijing National Laboratory for Condensed Matter Physics and Institute of Physics, Chinese Academy of Sciences, Beijing 100190, China; School of Physical Sciences, University of Chinese Academy of Sciences, Beijing 100190, China; Beijing National Laboratory for Condensed Matter Physics and Institute of Physics, Chinese Academy of Sciences, Beijing 100190, China; School of Physical Sciences, University of Chinese Academy of Sciences, Beijing 100190, China; Collaborative Innovation Center of Quantum Matter and School of Physics, Peking University, Beijing 100871, China; Beijing National Laboratory for Condensed Matter Physics and Institute of Physics, Chinese Academy of Sciences, Beijing 100190, China; Songshan Lake Materials Laboratory, Dongguan 523808, China; Songshan Lake Materials Laboratory, Dongguan 523808, China; Songshan Lake Materials Laboratory, Dongguan 523808, China; Beijing National Laboratory for Condensed Matter Physics and Institute of Physics, Chinese Academy of Sciences, Beijing 100190, China; School of Physical Sciences, University of Chinese Academy of Sciences, Beijing 100190, China; Beijing National Laboratory for Condensed Matter Physics and Institute of Physics, Chinese Academy of Sciences, Beijing 100190, China; School of Physical Sciences, University of Chinese Academy of Sciences, Beijing 100190, China; Beijing National Laboratory for Condensed Matter Physics and Institute of Physics, Chinese Academy of Sciences, Beijing 100190, China; School of Physical Sciences, University of Chinese Academy of Sciences, Beijing 100190, China; Beijing National Laboratory for Condensed Matter Physics and Institute of Physics, Chinese Academy of Sciences, Beijing 100190, China; School of Physical Sciences, University of Chinese Academy of Sciences, Beijing 100190, China; Beijing National Laboratory for Condensed Matter Physics and Institute of Physics, Chinese Academy of Sciences, Beijing 100190, China; School of Physical Sciences, University of Chinese Academy of Sciences, Beijing 100190, China; Beijing National Laboratory for Condensed Matter Physics and Institute of Physics, Chinese Academy of Sciences, Beijing 100190, China; School of Physical Sciences, University of Chinese Academy of Sciences, Beijing 100190, China; Beijing National Laboratory for Condensed Matter Physics and Institute of Physics, Chinese Academy of Sciences, Beijing 100190, China; Beijing National Laboratory for Condensed Matter Physics and Institute of Physics, Chinese Academy of Sciences, Beijing 100190, China; Songshan Lake Materials Laboratory, Dongguan 523808, China; Beijing National Laboratory for Condensed Matter Physics and Institute of Physics, Chinese Academy of Sciences, Beijing 100190, China; School of Physical Sciences, University of Chinese Academy of Sciences, Beijing 100190, China; Beijing National Laboratory for Condensed Matter Physics and Institute of Physics, Chinese Academy of Sciences, Beijing 100190, China; School of Physical Sciences, University of Chinese Academy of Sciences, Beijing 100190, China; Collaborative Innovation Center of Quantum Matter and School of Physics, Peking University, Beijing 100871, China; Beijing National Laboratory for Condensed Matter Physics and Institute of Physics, Chinese Academy of Sciences, Beijing 100190, China; School of Physical Sciences, University of Chinese Academy of Sciences, Beijing 100190, China; Beijing National Laboratory for Condensed Matter Physics and Institute of Physics, Chinese Academy of Sciences, Beijing 100190, China; School of Physical Sciences, University of Chinese Academy of Sciences, Beijing 100190, China; Songshan Lake Materials Laboratory, Dongguan 523808, China; Beijing National Laboratory for Condensed Matter Physics and Institute of Physics, Chinese Academy of Sciences, Beijing 100190, China; School of Physical Sciences, University of Chinese Academy of Sciences, Beijing 100190, China; Beijing National Laboratory for Condensed Matter Physics and Institute of Physics, Chinese Academy of Sciences, Beijing 100190, China; School of Physical Sciences, University of Chinese Academy of Sciences, Beijing 100190, China; Songshan Lake Materials Laboratory, Dongguan 523808, China

**Keywords:** 2D semiconductor, multilayer MoS_2_ wafer, layer-by-layer epitaxy, high performance transistors, thin film transistors

## Abstract

The 2D semiconductor of MoS_2_ has great potential for advanced electronics technologies beyond silicon. So far, high-quality monolayer MoS_2_ wafers have been available and various demonstrations from individual transistors to integrated circuits have also been shown. In addition to the monolayer, multilayers have narrower band gaps but improved carrier mobilities and current capacities over the monolayer. However, achieving high-quality multi-layer MoS_2_ wafers remains a challenge. Here we report the growth of high-quality multi-layer MoS_2_ 4-inch wafers via the layer-by-layer epitaxy process. The epitaxy leads to well-defined stacking orders between adjacent epitaxial layers and offers a delicate control of layer numbers up to six. Systematic evaluations on the atomic structures and electronic properties were carried out for achieved wafers with different layer numbers. Significant improvements in device performances were found in thicker-layer field-effect transistors (FETs), as expected. For example, the average field-effect mobility (*μ*_FE_) at room temperature (RT) can increase from ∼80 cm^2^·V^–1^·s^–1^ for monolayers to ∼110/145 cm^2^·V^–1^·s^–1^ for bilayer/trilayer devices. The highest RT *μ*_FE_ of 234.7 cm^2^·V^–1^·s^–1^ and record-high on-current densities of 1.70 mA·μm^–1^ at *V*_ds_ = 2 V were also achieved in trilayer MoS_2_ FETs with a high on/off ratio of >10^7^. Our work hence moves a step closer to practical applications of 2D MoS_2_ in electronics.

## Teaser

This work reports the first demonstration on the expitaxy of wafer-scale MoS2 multilayers with controlled number of layers and highest electronic qualities towards the practical electronic applications of 2D semiconductors.

## INTRODUCTION

Since the successful exfoliation of 2D MoS_2_ [1], these ultra-thin semiconductors have attracted great attention in the field of electronics [2–13]. Tremendous efforts have been devoted to exploring their scaled-up potentials, including both the wafer-scale synthesis of high-quality materials and application of them in large-area devices, with a specific focus on the monolayer MoS_2_ (ML-MoS_2_) [14–20]. Until now, high-quality ML-MoS_2_ wafers have been available from various growth approaches including chemical vapor deposition (CVD) [15–20] and metal-organic CVD (MOCVD) [14]. Depending on the growth approaches and substrates, the MOCVD/CVD ML-MoS_2_ films are generally stitched from random/aligned domains with sizes featured at the 1/100 micron level and have a state-of-the-art room temperature electron mobility of ∼30/∼70 cm^2^·V^–1^·s^–1^ on average—an electronic quality comparable with or even better than the exfoliated monolayers.

In terms of a further improvement of the electronic quality of the large-scale 2D-MoS_2_, structural imperfections should be eliminated as much as possible; however, there is not much space left for monolayer MoS_2_ after 10 years of synthesis optimizations in this field. Another direction is to switch to multi-layer MoS_2_, e.g. bilayers or trilayers, since they have intrinsically higher electronic quality than monolayers [21–28]. Indeed, with the increased number of MoS_2_ layers, decreased band gaps but enhanced electron mobilities and current densities have been demonstrated in exfoliated or CVD flakes [21,27,28]. However, it currently remains a significant challenge to produce high-quality and large-scale MoS_2_ multilayers with a well-controlled number of layers. Previously, CVD and sulfurization have been used to produce multi-layer MoS_2_ in the form of flakes. While those flakes are of good crystal quality, their sizes are small, at typically less than ∼300 μm [25,29]. Large-scale multi-layer MoS_2_ films have been also synthesized, e.g. from sulfurization of precoated Mo/MoO_3_ films [30] and atomic layer deposition (ALD) [31]. As-produced films are typically polycrystalline with many randomly oriented domains in sizes of <100 nm and include the co-existence of different layer thicknesses. Such poor crystalline quality, subjected to bad domain stitching and less control on the number of layers, leads to low electronic performances that are even worse than those achieved in MoS_2_ monolayers [32–35]. More details appear in the Supplementary Information (Supplementary Tables S1 and S2).

Generally, to produce MoS_2_ multilayers, the best practice is to begin with monolayers and then increase their thicknesses by gradually growing additional layers. However, considering the case of free-standing MoS_2_, this route is problematic from the thermodynamic point of view. The surface energy of free-standing MoS_2_ increases with the number of layers [36,37]; it is thus energetically unfavorable to increase additional layers [38]. This fundamental thermodynamic limitation has likely prevented large-area multi-layer MoS_2_ with well-controlled layer numbers from being demonstrated previously. It is expected that this thermodynamic limitation might be overcome by engineering the surface energy of MoS_2_ via the proximity effect. According to our density functional theory simulations, surface energies of mono- and bilayer MoS_2_ on a sapphire (0001) surface are significantly elevated, making the growth of an additional layer on top of them thermodynamically feasible. More details and discussions appear in the Supplementary Information.

**Figure 1.**
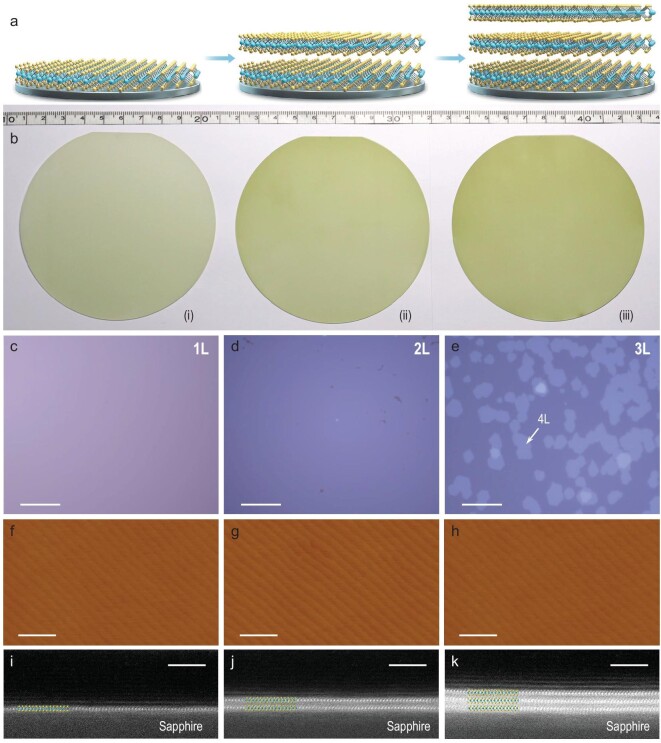
Layer-by-layer epitaxy of multi-layer MoS_2_ wafers. (a) Schematic illustration of epitaxy process. (b) Photographs of 4-inch MoS_2_ wafers: (i) monolayer, (ii) bilayer, (iii) trilayer. (c–e) Optical images of wafers shown in (b). Quadrilayer domains on the trilayer film are marked by a representative white arrow. Scale bars: 30 μm. (f–h) AFM amplitude images taken from mono-, bi- and trilayer wafers. Scale bars: 500 nm. (i–k) Cross-sectional HAADF-STEM images of epitaxial mono-, bi- and trilayer MoS_2_. Scale bars: 3 nm.

In this work, we developed a new technique, i.e. layer-by-layer epitaxy, to grow high-quality 4-inch multi-layer MoS_2_ wafers with a controlled number of layers. By using sapphire (0001) as the starting substrate, we successfully achieved the growth of uniform NL-MoS_2_ (*N* = 1, 2, 3), where *N* is the number of layers, in a layer-by-layer manner. All sapphire wafers used in our growth are 4-inch wafers cut along the zero-degree plane, or C-Cut, which were vacuum annealed at ∼1000ºC to form atomically flat surfaces before epitaxy. Note that sapphire wafers are cheap and widely used for various semiconductor thin-film epitaxy, and the sapphire (0001) surface is so far one of the best substrates for MoS_2_ epitaxy due to a negligible lattice mismatch. Two processes are involved in this layer-by-layer growth, i.e. heteroepitaxy of the first layer on sapphire and homoepitaxy of the (*N* + 1)th layer on NL with *N* > 0, as illustrated in Fig. 1a.

## RESULTS AND DISCUSSION

Both heteroepitaxy and homoepitaxy growth were performed in a multi-source oxygen-enhanced CVD system. This new CVD approach for monolayer MoS_2_ growth features a greatly enhanced growth rate and excellent film uniformity across the entire 4-inch sapphire surface (benefitting from the stable and uniform S-source and Mo-source supply during the growth process) [17]. Usually, the first-layer epitaxy on sapphire starts from nucleation at multiple sites, proceeds with the edge growth of those nuclei and eventually reaches a layer completion (i.e. full coverage on the substrate surface) via the domain–domain coalescence mechanism. Typically, the first-layer growth lasts for 30 min and a completed layer is stitched from two kinds of triangular domains inversely aligned along sapphire <11–20>, as illustrated in Supplementary Fig. S6.

Note that monolayer MoS_2_ growth on sapphire or SiO_2_ substrates follows a unique self-limiting process [15] in which additional layers can hardly be nucleated on the monolayer until its completion. After the first-layer completion, we can grow additional layers epitaxially on top of the first layer by using this oxygen-enhanced CVD technique. One key process is to control the nucleation density of the second layer (more discussions appear in the Supplementary Information). A higher temperature of the Mo-source (*T*_Mo_)—in other words, a higher Mo-source flux—is found to be beneficial to achieving a higher nucleation density of the second layer. We thus increased both *T*_Mo_ and the substrate temperature (*T*_substrate_) to enable dense nucleation of the second layer to reach a saturation state under which additional layer nucleations are forbidden (see Supplementary Information for more discussions). As shown in Supplementary Fig. S7, the second-layer nucleations are dense and uniform across the entire 4-inch surface. In a similar way as mentioned above, second-layer nuclei grow then stitch for the layer completion, yielding a continuous and fully covered bilayer MoS_2_ film eventually, as demonstrated in Supplementary Fig. S5. From Supplementary Fig. S5, we can see that domains in the first layer are triangular with sizes of ∼200 μm on average, whereas in the second layer, they are hexagonal with sizes reduced to ∼10 μm on average. Note that the domain shape is defined by the growth rate at the Mo-terminated (*V*_Mo_) and S-terminated edges (*V*_S_), and the hexagonal shape corresponds to *V*_Mo_ ≈ *V*_S_ [39]; we intentionally modulated the shape of the second domains to be hexagonal, as the hexagonal shape is beneficial for better domain–domain stitching from a geometrical point of view. After completing the second layer, we thus can repeat the homoepitaxy process by prolonging the growth time to achieve fully covered multilayers with controlled *N* in a layer-by-layer manner. A detailed sequential process for 3L-MoS_2_ on sapphire is illustrated in Supplementary Fig. S6.

As shown above, the dedicated control of the growth kinetic process, e.g. nucleation and edge growth, is the key to achieving continuous layer epitaxy. In our growth tests, we achieved MoS_2_ wafers with *N* up to 6 (Supplementary Fig. S8). It was noticed that the ideal 2D growth mode is difficult to keep the *N*th layer when *N* ≥ 3, leading to the appearance of additional mono- or multi-layer domains on NL-/MoS_2_ (refer to Supplementary Fig. S8). Such a failure is more and more significant with increasing *N* and the growth mode evolves gradually from 2D to 3D, in consistence with the classical Stranski–Krastanov growth mode [40]. This layer-dependent growth mode evolution could be attributed to several reasons. First, the surface proximity effect reduces quickly for those thicker layers with upper surfaces farther away from the sapphire surface. Besides, once the additional layers appear, their presence would be amplified in the subsequent growth. More detailed discussions on energetics, growth kinetics and analysis of practical growth parameters appear in the Supplementary Information.

Since as-grown MoS_2_ films are very uniform for mono-/bilayers and quite uniform for trilayers (as characterized in Supplementary Fig. S9) across entire 4-inch wafers, we thus mainly focus on the bilayer and trilayer samples in the following characterizations. Figure 1b shows typical optical images of the as-grown 4-inch mono-, bi- and trilayer MoS_2_ wafers. Figure 1c–h shows typical zoom-in optic and atomic force microscope (AFM) images from these wafers, indicating the full coverage and very clean surfaces. The trilayer continuous films have certain additional small quadrilayer domains and their coverage is ∼30%. The layer numbers were further confirmed by high-resolution cross-section high-angle annular dark-field scanning transmission electron microscopy (HAADF-STEM) imaging (Fig. 1i–k). We can see clearly that each layer consists of one-layer Mo and two-layer S atoms with a layer thickness of ∼0.62 nm and the interface between the adjacent layers is atomically clean and sharp, reflecting the superiority of epitaxy.

**Figure 2.**
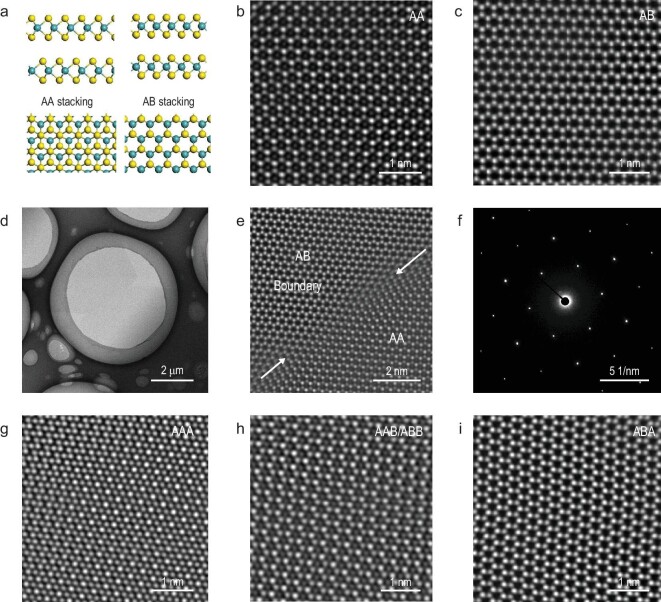
Stacking configurations in the epitaxial multi-layer MoS_2_. (a) Side and top views in ball-and-stick mode of the atomic structures for AA-stacked and AB-stacked MoS_2_ bilayer. (b and c) STEM images of AA-stacked and AB-stacked bilayer MoS_2_, respectively. (d) STEM image of two emerged flakes with AA and AB stacking orders_._ (e and f) STEM and SAED images of the boundary area shown in (d). (g–i) STEM images of the AAA-stacked (g), AAB/ABB-stacked (h) and ABA-stacked (i) trilayer MoS_2_.

To elucidate the layer stacking orders in these multi-layer MoS_2_ wafers, we further performed atomic structure characterizations by STEM. As shown in Fig. 2, there are two stacking orders in our bilayer samples, i.e. AA stacking (2L-AA, 3R phase) and AB stacking (2L-AB, 2H phase), and the corresponding atomic configurations are shown in Fig. 2a. Figure 2b and c shows STEM images of a typical AA-stacked and AB-stacked bilayer MoS_2_. Note that the AA-stacked layers have no inversion symmetry while the AB-stacked layers have. Figure 2d and e also shows the STEM images of a bilayer MoS_2_ film with a grain boundary. AA-stacked and AB-stacked domains can be distinguished and these two different stacking domains can coalesce together without any disconnect gap, revealing a crystalline continuity. Figure 2f shows the selected area electron diffraction (SAED) pattern at the grain boundary area, exhibiting only one set of hexagonal diffraction spots, as expected. We also characterized the trilayer samples. Differently from the bilayer case, the stacking orders in trilayers are much more complicated (Supplementary Fig. S10). AAA, AAB/ABB and ABA stacking configurations all exist, as shown in Fig. 2g–i. All these STEM images for bi- or trilayers reveal our epitaxial multi-layer films having excellent lattice alignments. Benefitting from the epitaxy technique, the seamless stitching of these aligned domains leads to high crystalline quality of multi-layer MoS_2_ on sapphire, as will be confirmed by our latter device characterizations.

Second-harmonic generation (SHG) microscopy was also performed to further study the large-scale stacking orders in our bilayer films due to the distinct intensity difference between AA-stacked and AB-stacked structures [33]. Note that the AA bilayers have stronger SHG intensities than monolayer MoS_2_ crystals due to the broken inversion symmetry, whereas the AB bilayers have weak SHG intensities due to the restored inversion symmetry [41]. As shown in Supplementary Fig. S11a, the SHG mapping image of ∼1.7 L MoS_2_ shows obvious contrast of monolayers, 2L-AA and 2L-AB layers. Supplementary Fig. S11b shows the SHG mapping image of our bilayer continuous films; it shows two main contrasts with monolayer and trilayer areas barely seen, which confirms that our bilayer films consist of two stacking orders.

**Figure 3.**
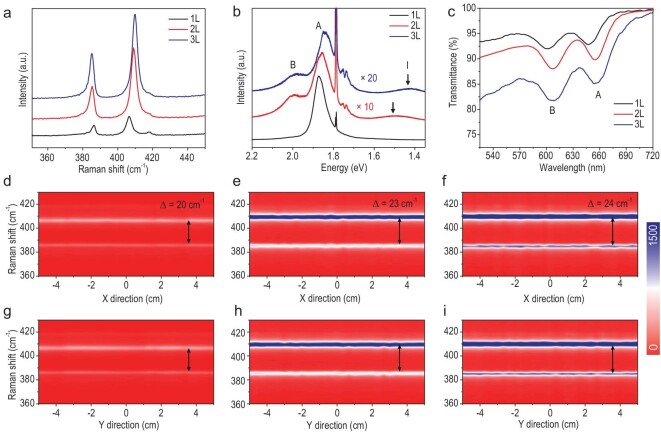
Spatial uniformity of multi-layer MoS_2_ wafers. (a–c) Raman, PL and transmittance spectra of the as-grown mono-, bi- and trilayer MoS_2_ wafers. (d–i) Color-coded images of typical Raman line scan mapping along the horizontal and longitudinal direction of (d and g) monolayer, (e and h) bilayer and (f and i) trilayer MoS_2_ wafers. Each line scan along either the X-direction or Y-direction of the wafer includes 31 data points.

As mentioned above, MoS_2_ multilayers would have *N*-dependent band gaps. To confirm this in our epitaxial samples, we thus collected optical spectra for our mono, bi- and trilayer MoS_2_ wafers. Corresponding Raman spectra are shown in Fig. 3a. In control samples of monolayer MoS_2_ films, the peak frequency difference (Δ) between the E_2g_ and A_1g_ vibration modes is ∼20 cm^–1^. As a comparison, Δ in the bilayer and trilayer films are wider, at ∼23 and ∼24 cm^–1^, respectively. Figure 3b shows the photoluminescence (PL) spectra of our mono-, bi- and trilayer MoS_2_ films. We can see a strong A-exciton peak at ∼1.88 eV in the monolayer, while A-exciton and B-exciton peaks are greatly suppressed in bi- and trilayer films due to the transition from the direct band gap to the indirect ones [42,43]. The indirect band gaps are ∼1.50 and ∼1.42 eV for bilayers and trilayers, respectively, confirming the *N*-dependent band gaps of the multi-layer MoS_2_. Note that those sharp peaks at 1.79 eV are from sapphire substrates. Figure 3c shows the optical transmittance spectra of mono-, bi- and trilayer MoS_2_ films transferred on quartz substrates and the corresponding transmittances are 94.2%, 91.6% and 84.5% at a wavelength of ∼550 nm. Due to the release of the strain after the transfer, the A-exciton and B-exciton peaks in the transmittance spectra are slightly shifted. Using the Raman line scanning, we also investigated the wafer-scale uniformity of the as-grown mono-, bi- and trilayer MoS_2_ wafers, as shown in Fig. 3d–i. We can see that these Raman peaks locate nearly the same along the entire wafer diameter, revealing a high uniformity.

**Figure 4.**
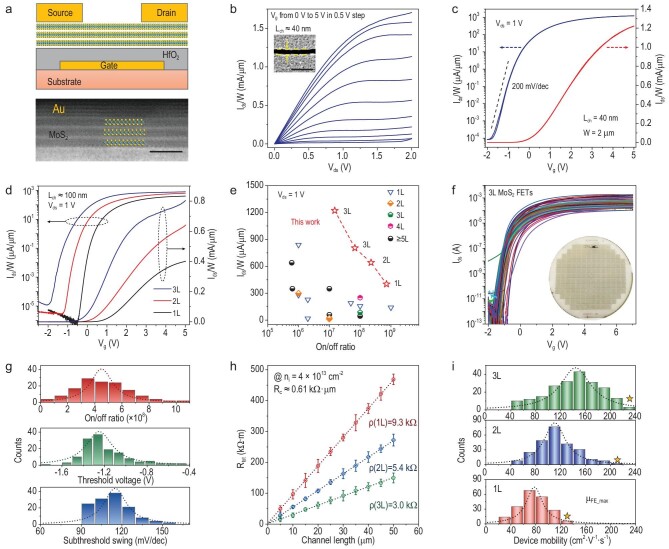
Benchmark testing of multi-layer MoS_2_ FETs. (a) Schematic view (top) of back-gated MoS_2_ FET and cross-section STEM image (bottom) of a trilayer FET at the MoS_2_–Au contact region. Scale bar: 1 nm. (b and c) Typical output/transfer curves of a trilayer MoS_2_ FET. *L*_ch_ = 40 nm, *t*_HfO2_ = 5 nm. Inset to (b) shows the SEM image of the channel. Scale bar: 200 nm. (d) Comparison of transfer curves of mono-, bi- and trilayer MoS_2_ FETs with *L*_ch_ ≈ 100 nm. (e) The comparisons of current densities (@*V*_ds_ = 1 V) and on/off ratios with previous works. The detailed device parameters are shown in Supplementary Table S1. (f) Transfer curves of 150 trilayer MoS_2_ FETs at *V*_ds_ = 1 V. *L*_ch_ = 5–50 μm, *t*_HfO2_ = 10 nm. Inset to (f) shows photograph of wafer-scale MoS_2_ FET array. (g) Statistical distribution of on/off ratio (red), threshold voltage (green) and subthreshold swing (blue) from the 150 trilayer MoS_2_ FETs. (h) The sheet resistance *ρ* and contact resistance *R*_c_ extracted from mono-, bi- and trilayer MoS_2_ FETs. (i) Statistical distribution of device mobility of mono-, bi- and trilayer MoS_2_ FETs. The yellow stars indicate the maximum values achieved in each type of device.

Based on the obtained high-quality multi-layer MoS_2_ wafers, we hence fabricated field-effect transistors (FETs) for performance benchmark testing. Please see ‘Methods’ and Supplementary Fig. S12 for details on device fabrications. Let us look at the short-channel trilayer MoS_2_ FETs first. The structure of these back-gated MoS_2_ FETs is illustrated in Fig. 4a. High-resolution STEM imaging at the MoS_2_–Au interface (as illustrated in the bottom image of Fig. 4a) reveals a sharp contact interface without obvious damage, filamentous breaks or wrinkles [8,44–46]. The output and transfer curves of a device with a channel length (*L*_ch_) of 40 nm are shown in Fig. 4b and c. Linear output characteristics at small bias voltages (*V*_ds_) suggest the ohmic contact behavior and the source–drain currents (*I*_ds_) quickly approach to saturation at small gate voltages subjected to the employment of a HfO_2_ (*ϵ*_r_ = 15–20) dielectric layer. The device features a high on/off ratio of >10^7^, a sharp subthreshold swing (SS) of 200 mV·dec^–1^ over four magnitudes and a small hysteresis of Δ*V*_g_ ≈ 0.02 V (at 0.1 μA·μm^–1^). The current density (*I*_ds_/*W*, where *W* is the channel width) can reach 1.70/1.22/0.94 mA·μm^–1^ at *V*_ds_ = 2/1/0.65 V, which is the highest ever achieved in MoS_2_ transistors. Such high on-current density is above the target of high-performance logic transistors from the International Roadmap for Devices and Systems (IRDS) 2024. The transfer curve of the *L*_ch_ = 40 nm trilayer FET at *V*_ds_ = 0.65 V is shown in Supplementary Fig. S13.

Transfer curves of mono-, bi- and trilayer devices with *L*_ch_ = 100 nm are shown in Fig. 4d. We can see a significant improvement in the on-current densities while increasing the number of layers and the corresponding *I*_ds_/*W* of mono-, bi- and trilayer devices are 0.40, 0.64 and 0.81 mA·μm^–1^, respectively, at *V*_ds_ = 1 V and *V*_g_ = 5 V (Supplementary Fig. S14). It was also noted that thicker MoS_2_ devices show saturated currents at much smaller *V*_g_. In Fig. 4e, we plotted the current densities (*V*_ds_ = 1 V) and on/off ratios of our devices compared with previous data from the state-of-the-art MoS_2_ devices (refer to Supplementary Table S3 for more details). The good balance between high current density and high on/off ratio suggests great potential for these epitaxial multi-layer MoS_2_ wafers for in the fabrication of integrated, high-performance and low-power electronics.

Next, we also fabricated long-channel FETs with *L*_ch_ varying from 5 to 50 μm and *W*_ch_ varying from 10 to 30 μm based on our multi-layer MoS_2_ wafers, as illustrated in the inset of Fig. 4f. Transfer curves of 150 randomly picked trilayer MoS_2_ FETs with different *L*_ch_ and *W*_ch_ are shown in Fig. 4f (similar data from mono- and bilayer MoS_2_ FETs can be found in Supplementary Fig. S15). We also show transfer curves of 100 randomly picked trilayer MoS_2_ FETs with the same *L*_ch_ = 10 μm and *W*_ch_ = 10 μm in Supplementary Fig. S16. The overall yield of all devices is >95%. All these devices exhibit small device-to-device variations, reflecting the uniformity of epitaxial wafers. On/off ratios, subthreshold voltages (*V*_th_) and SS of these devices are also plotted in Fig. 4g. The highest on/off ratio can reach to 10^8^–10^9^ and averages at 4.5 × 10^8^, which is much higher than that achieved in previous multi-layer MoS_2_ devices [32,35,47]. *V*_th_ is mainly located at –1.25 ± 0.4 V and the average SS is ∼115 mV/dec.

Finally, let us compare the film conductivities of mono-, bi- and trilayer MoS_2_. The sheet resistances (*ρ*) were extracted using the transfer length method (TLM) [48] as shown in Fig. 4h. At a carrier density of n_i_ ≈ 4 × 10^13^ cm^–2^, *ρ* is 9.3, 5.4 and 3.0 kΩ for mono-, bi- and trilayer MoS_2_ channels, respectively, revealing that multi-layer MoS_2_ is more conductive. Besides, the extracted contact resistance (*R*_c_) is ∼0.61 kΩ·μm at *n*_i_ ≈ 4 × 10^13^ cm^–2^. Although the achieved *R*_c_ is slightly larger than that of Bi-contacts reported recently [12], Au-contacts are advantageous considering that Au is stable and widely used nowadays in semiconductor technology. Better device performances might be achievable in the future by further optimizing contact techniques. In Fig. 4i, we summarize the field-effect mobilities (*μ*_FE_) of these long-channel MoS_2_ FETs. A significant improvement in *μ*_FE_ with channel layer numbers can be clearly seen. The average *μ*_FE_ is ∼80, ∼110 and ∼145 cm^2^·V^–1^·s^–1^ for mono-, bi- and trilayer FETs, respectively. The mobility distributions in each type of device are fitted by Lorentz curves. The full width at the half maximum (FWHM) of the fitting is ∼40, ∼50 and ∼60 cm^2^·V^–1^·s^–1^ for mono-, bi-, and trilayer devices, and the increased FWHM with the number of layers is partially attributed to the inhomogeneity from additional layers and need to be optimized in further studies. Remarkably, the highest *μ*_FE_ reaches 131.6, 217.3 and 234.7 cm^2^·V^–1^·s^–1^ in our mono-, bi- and trilayer devices, and all these numbers are record-high in wafer-scale MoS_2_ devices. Considering that, in well-developed thin-film transistors (TFTs), *μ*_FE_ is 10–40 cm^2^·V^–1^·s^–1^ for indium–gallium–zinc-oxide (IGZO) TFTs and 50–100 cm^2^·V^–1^·s^–1^ for low-temperature polycrystalline silicon (LTPS) TFTs [49], the competitive average *μ*_FE_, i.e. >100 cm^2^·V^–1^·s^–1^, achieved in this work also reveals great potential for these multi-layer MoS_2_ films for TFT applications.

## CONCLUSION AND PERSPECTIVE

As shown above, the developed layer-by-layer epitaxy on sapphire can yield uniform and large-scale multi-layer MoS_2_ with clean interfaces and a well-controlled number of layers, e.g. one, two and three. In each layer, high lattice continuity/quality is accomplished via the seamless stitching of large domains aligned along sapphire <11–20>. Bilayer and trilayer MoS_2_ wafers exhibit remarkably improved electrical quality over their monolayer counterparts, as evidenced by higher on-current densities and higher electron mobilities, suggesting great potential for using them in 2D electronics. Regarding technological improvements, further investigations are required. First, the high-temperature growth process is less compatible with conventional semiconductor processes and thus needs to be lowered. Second, steady improvements in wafer sizes and control of the single alignment of domains are also required for producing single-crystalline multilayers on a large scale. Besides, it is also very interesting to apply this layer-by-layer epitaxy technique for large-scale and high-quality heterogeneous 2D layers to broaden the application field of 2D semiconductors.

## METHODS

### Layer-by-layer epitaxy of MoS_2_

All growths were carried out in a home-built multi-source CVD system with three temperature zones, named zone-I, zone-II and zone-III. In a typical growth, one S-source (Alfa Aesar, 99.9%, 15 g) was loaded into zone-I and carried by Ar (40 sccm) and six MoO_3_-source (Alfa Aesar, 99.999%, 30 mg each) were loaded into zone-II and carried by Ar/O_2_ (40/1.7 sccm) individually. Sapphire substrates (single side polished, c-plane (0001) with off-set angle (*M*-axis) of 0.2 ± 0.1 deg., 4-inch wafers) were loaded into zone-III. During the heteroepitaxy of MoS_2_ on sapphire, the temperature in zone-I, zone-II and zone-III was kept at 120ºC, 540ºC and 910ºC, respectively, while the temperature in zone-II and zone-III was increased to 570ºC and 940ºC, respectively, for homoepitaxy of MoS_2_.

### Structural and spectroscopic characterizations

AFM imaging was performed using the Asylum Research Cypher S system. Raman and PL spectra were collected using the Horiba Jobin Yvon LabRAM HR-Evolution Raman system with an excitation laser wavelength of 532 nm. SAED was performed in a STEM (JEOL Grand ARM 300 CFEG) operating at 80 kV and atomic-resolution images were achieved using an aberration-corrected scanning transmission electron microscope Grand ARM 300 (JEOL) operating at 80 kV.

### SHG measurements

The SHG mapping was recorded using a home-built confocal microscope. The 1200-nm pulsed laser (100 fs, 76 MHz) was generated using a Ti:sapphire oscillator (Coherent Mira-HP) equipped with an optical parametric oscillator (Coherent Mira-OPO-X). The laser beam was sent through a linear polarizer followed by a half-wave plate to tune the polarization direction. Then the laser beam was focused on the sample at normal incidence by the objective (40×, Numerical aperture = 0.65). In the reflection geometry, the parallel component of SHG from the sample was extracted using a linear analyser parallel to the incident polarization. The SHG signal at each point of the sample was recorded using a grating spectrograph with a charge-coupled device camera (Princeton SP-2500i).

### Device fabrications and measurements

FETs were fabricated using the lithography and etching process. The device fabrication process is illustrated in Supplementary Fig. S6. First, buried back-gates of Ti/Au/Ti (1/5/1 nm) were patterned on substrates using lithography and e-beam evaporation at a deposition rate of 0.01–0.05 Å/s. Second, HfO_2_ with a thickness of 5–15 nm was deposited using ALD (Savannah-100 system, Cambridge NanoTech. Inc. Precursors: H_2_O and tetrakis dimethylamino hafnium; deposition temperature: 200ºC) as the gate dielectric layer. Third, MoS_2_ films were etched off from sapphire substrates in KOH solution (1 mol/L) at 110ºC and transferred onto the as-prepared HfO_2_/metal-gate/sapphire surfaces. After the transfer, lithography and oxygen plasma etching (Plasma Lab 80 Plus, Oxford Instruments Company) were used to define the MoS_2_ channel region. Finally, e-beam evaporated Au (20 nm) was deposited for the source–drain contact metal. For short-channel (*L* < 100 nm) FETs, the substrate was SiO_2_ and the channels were defined using standard e-beam lithography (Raith e-Line plus system) using PMMA (495 A2) as the resist layer (spin-coated at 2000–3000 rpm and baked at 180ºC for 2 min). For long-channel (L > 2 μm) FETs, the substrate was sapphire and the channels were defined using UV-lithography (MA6, Karl Suss) with AR-P 5350 (ALLRESIST GmbH) as the positive photoresist with a thickness of ∼1 μm (spin-coated at 4000 rpm and baked at 100ºC for 4 min). Note that we also used oxygen plasma to clean the photoresist residues before depositing the Ti/Au/Ti back-gate electrodes before ALD. All electrical measurements were carried out in a four-probe vacuum station (base pressure: ∼10^−6^ mbar) equipped with a semiconductor parameter analyser (Agilent B1500).

## Supplementary Material

nwac077_Supplemental_File
