# Supplementary material for: Layer-by-layer epitaxy of multi-layer MoS2 wafers
Source: Natl Sci Rev. 2022 Apr 21;9(6):nwac077. doi: 10.1093/nsr/nwac077 (PMC9232293; doi:10.1093/nsr/nwac077)
Supplement: nwac077_Supplemental_File [file nwac077_supplemental_file.pdf]

Supplementary Information for

Layer-by-Layer Epitaxy of Multilayer MoS_2 Wafers

Qinqin Wang[1,2†], Jian Tang[1,2†], Xiaomei Li[1,2], Jinpeng Tian[1,2], Jing Liang[4], Na Li[1,3], Depeng Ji[3], Lede Xian[3], Yutuo Guo[1,2], Lu Li[1,2], Qinghua Zhang[1,2], Yanbang Chu[1,2], Zheng Wei[1,2], Yanchong Zhao[1,2], Luojun Du[1], Hua Yu[1,3], Xuedong Bai[1,2], Lin Gu[1,2], Kaihui Liu[4], Wei Yang[1,2], Rong Yang[1,2,3], Dongxia Shi[1,2] & Guangyu Zhang[1,2,3*]

[1] Beijing National Laboratory for Condensed Matter Physics and Institute of Physics, Chinese Academy of Sciences, Beijing 100190, China

[2] School of Physical Sciences, University of Chinese Academy of Sciences, Beijing 100190, China

[3] Songshan Lake Materials Laboratory, Dongguan 523808, China

[4] Collaborative Innovation Center of Quantum Matter and School of Physics, Peking University, Beijing 100871, China

† Authors contribute equally to this work.

* Corresponding author. Email: gyzhang@iphy.ac.cn

Contents:

PART 1: Additional Discussions on the Growth and Results

PART 2: Additional Characterization Data and Illustrations

PART 3: References

PART 1: Additional Discussions on the Growth and Results

I: Energetics of layer-dependent MoS_2 on sapphire

Generally, three scenarios are used to describe the epitaxial thin film growth on a substrate: I) Frank-van der Merwe (FM) growth mode (2D growth mode); II) Volmer-Weber (VW) growth mode (3D growth mode); and III) Stranski-Kranstanov growth mode (initially 2D, after a critical thickness, it evolves to 3D growth mode). Regarding thermodynamics, these different modes are governed by three thermodynamic quantities, i.e. the free energy per unit area at the overlayer-vacuum interface (γ_o), the overlayer-substrate interface (γ_i), and the substrate-vacuum interface (γ_s). According to criteria from E. Bauer & J. H. van der Merwe[1], if $\Delta\gamma=\gamma_o+\gamma_i-\gamma_s < 0$, the growth mode is 2D; and if $\Delta\gamma \geq 0$, the growth mode is 3D. Where $\Delta\gamma$ can be understood as the energy cost per unit area for the lateral growth of the overlayer (see Fig. S1a).

For free-standing MoS_2, now we consider the case of growing an additional layer on (N-1)L-MoS_2. As for free-standing MoS_2, the interfacial energy γ_i =0, the above energy criteria is simply reduced to the surface energy difference between NL-MoS_2 and (N-1)L-MoS_2. According to previous studies[2], the surface energy (γ_N) increases with the number of layers (N) from ~0.23 J/m^2 (N=1) to ~0.26 J/m^2 (N>10, bulk). As $\gamma_{N-1}<\gamma_N$, thus $\Delta\gamma>0$, suggesting that this process is thermodynamically unfavorable. Our DFT calculations also confirm such results (see Fig. S1d).

To overcome this limitation, one expected route is to utilize the surface proximity effect by putting NL-MoS_2 on substrate with high surface energy. Since the surface energy of sapphire (0001) is very high, ~3.3 J/m^2,[3] we now look at the NL-MoS_2/sapphire system from DFT calculations. DFT calculations are carried out with the Vienna *ab initio* Simulation Package (VASP)[4]. The projector augmented wave (PAW) method[5] and generalized gradient approximation with Perdew, Burke and Ernzerhof type of (GGA-PBE) pseudopotentials[6] are used.

We can adapt the above criteria to evaluate the energetics of 2D versus 3D growth of MoS_2 on sapphire. For the growth of monolayer MoS_2 on sapphire, we can use the same energy criteria $\Delta\gamma$ as defined above. However, for the growth of the Nth layer on N-1 layer, we need to rewrite the energy criterion as:

$$\Delta\gamma_N=\gamma_{oN} + \gamma_{iN} - (\gamma_{oN-1} + \gamma_{iN-1}), \quad (1)$$

where $\gamma_{o\alpha}$ is the surface energy of the α-layer MoS_2 on sapphire and $\gamma_{i\alpha}$ is the interfacial energy at the α-layer MoS_2 - sapphire interface. This is because now the bare region, or the "substrate", becomes the (N-1)-layer MoS_2 on sapphire (see Fig. S1b).

We can use the total energy of the N/(N-1)-layer MoS_2 on sapphire systems to calculate $\Delta\gamma_N$. As shown in Fig. S1b, the total energy per unit area of N-layer MoS_2 on sapphire can be written as:

$$E_N = E^{bulk}_{sapphire}d + NE^{bulk}_{MoS_2} + \gamma_{oN} + \gamma_{iN} + \gamma_s, \quad (2)$$

where $E^{bulk}_{sapphire}$ and $E^{bulk}_{MoS_2}$ are the total energy of bulk sapphire per unit thickness and bulk MoS_2 per layer, respectively; d is the thickness of the sapphire substrate; and γ_s is the surface energy of the other surface of sapphire without MoS_2. Similarly, we can write down the total energy per unit area of (N-1)-layer MoS_2 on sapphire as:

$$E_{N-1} = E^{bulk}_{sapphire}d + (N-1)E^{bulk}_{MoS_2} + \gamma_{oN-1} + \gamma_{iN-1} + \gamma_s. \quad (3)$$

From Eqn. (1-3), we have

$$\Delta\gamma_N = E_N - E_{N-1} - E^{bulk}_{MoS_2}. \quad (4)$$

The layer-dependent relative energies $\Delta\gamma_N$ of growing the Nth layer MoS_2 on (N-1)L-MoS_2/ sapphire calculated with DFT are shown in Fig. S1c. From these data, we can see that $\Delta\gamma < 0$ criterion is satisfied when N=1, 2, while $\Delta\gamma$ is close to 0 when N=3, and $\Delta\gamma > 0$ when N>3. These results suggest that the epitaxial growth of an additional MoS_2 layer on 1L-MoS_2/sapphire (to produce bilayer) or 2L-MoS_2/sapphire (to produce trilayer) follows the 2D growth mode; for epitaxy on thicker MoS_2, the growth evolves from 2D to 3D at a critical thickness, e.g., N=3.

From these results shown in Fig. S1(c-d), we can clearly see the behaviors of additional layer of MoS_2 grown on freestanding (N-1)L-MoS_2 and (N-1)L-MoS_2/sapphire are distinctly different for small N, although both cases are homoepitaxy. While the layer-by-layer homoepitaxial growth of MoS_2 in the free-standing scenario is energetic unfavorable, such 2D growth is energetic favorable for the first few layers of MoS_2 grown on sapphire surface, due to the proximity effect of the sapphire surface that has a very high surface energy.

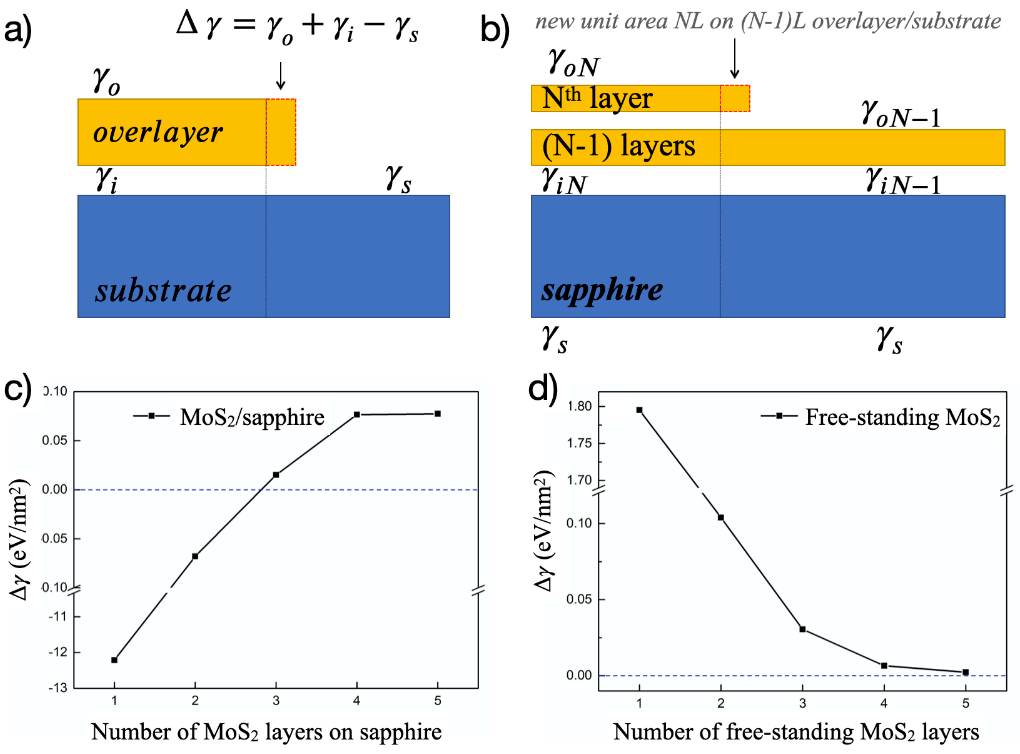

Fig. S1 Energetics of layer-dependent MoS_2 with and without sapphire substrate. (a) Model of an overlayer grown on a substrate. (b) Model of Nth monolayer epitaxially grown on (N-1)L-MoS_2/sapphire. (c-d) The calculated layer-depended relative surface/interfacial energies of MoS_2/sapphire and free-standing MoS_2.

II: Kinetics of the layer-by-layer growth of MoS_2

Above analysis provides a thermodynamic validation of layer-by-layer growth of MoS_2 on sapphire. Here, we further discuss in view of kinetics. Generally, the epitaxy (such as MBE) of a complete thin layer on substrate starts from multiple nucleations. These nuclei grow into larger domains at their edges (edge growth) and eventually coalesce into a complete film. In the present case, the general description of $(N+1)^{th}$L-MoS_2 on NL-MoS_2/sapphire is illustrated in the below model (Fig. S2). N=0 (N>0) corresponds heteroepitaxy (homoepitaxy).

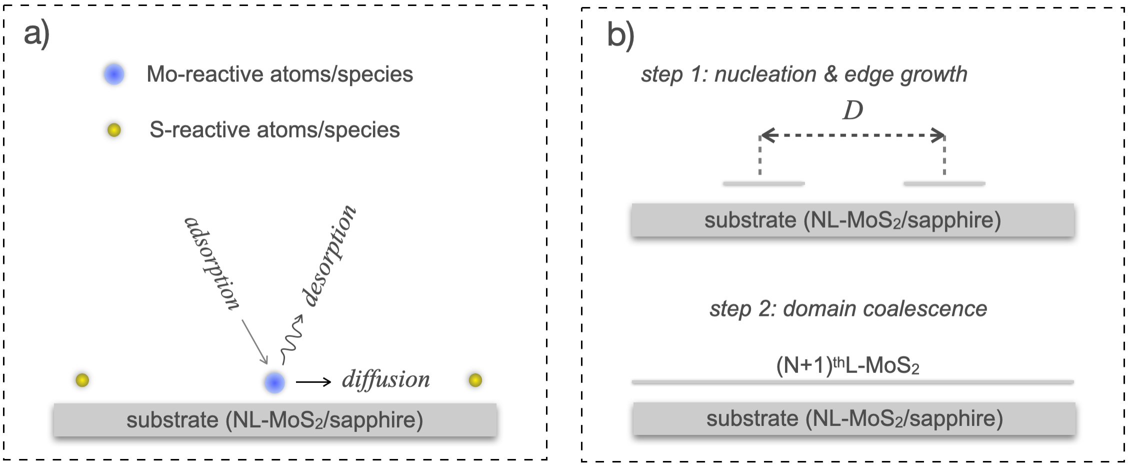

Fig. S2 Kinetics and model for layer-by-layer growth MoS_2.

In this model, surface diffusions are mainly considered. During the growth, reactive Mo-/S- atoms/species reach to the substrate surface. Three processes are involved (Fig. S2a): 1) the adsorption on the surface; 2) the diffusion on the surface with a lifetime before de-adsorption; and 3) the desorption off the surface. The light element S desorbs easily off the surface at high growth temperatures (with very small τ_S); thus, we could mainly consider the kinetic behavior of Mo-atoms/species which dominates the growth, as confirmed from many previous results. The diffusion behavior of these reactive atoms/species is also surface dependent, e.g. between sapphire and MoS_2. According to a control experiment shown in below Fig. S3, these reactive atoms/species are much more diffusive on MoS_2 than sapphire. Such high diffusivity can be attributed to the atomic smooth and dangling bonds free surfaces of the MoS_2 basal plane, and it will be even more significant when the substrate temperature is high (such as >1000 K). We also assume that, when N>0, the diffusion behavior is N-independent under the same experimental conditions since all surfaces are of MoS_2.

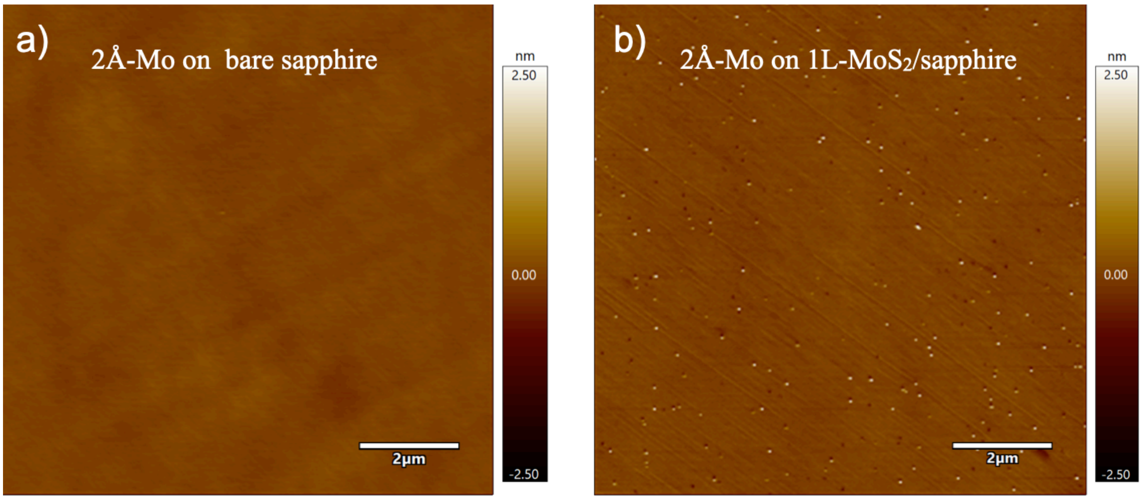

Fig. S3 AFM images of 2Å-thick Mo on a) bare sapphire and b) 1L-MoS_2/sapphire surfaces. Both Mo-deposition and AFM imaging were performed at a room temperature. Results suggest that, even at room temperature, Mo atoms are very diffusive on the MoS_2 surface and aggregate into small particles. In contrast, Mo on sapphire surface is quite stable at room temperature.

A growth cycle of a complete $(N+1)^{th}$L is depicted in Fig. S2b, involving two steps. At the initial stage in step 1, Mo-/S- atoms/species meet on the surface of NL-MoS_2/sapphire and react for $(N+1)^{th}$L nucleation. Here only monolayer nucleation is considered for simplicity (more discussions are given in the following Part-**III**). After the initial nucleation, the growth faces a balance between new nucleations and the edge growth. If the edge growth rate is too fast, those initial nuclei will

quickly grow and cover the entire substrate by domain-domain coalescence (step 2); in this case (defined as case 1), nucleations proceed over the entire growth cycle. Otherwise (defined as case 2), the density of surface nuclei would increase with time till reaching saturation. The saturation density is characterized by a critical nucleation time t_C after which the formation of new nuclei ceases (when the density of stable nuclei is high enough so that the nearby atoms/species would diffuse to existing nuclei rather than forming new ones). After the nucleation saturation, only the edge growth is present and eventually achieve layer completion by domain-domain coalescence (step 2). As will be discussed in the following Part-**III**, case 1/2 corresponds to the hetero-/homo-epitaxy process.

Obviously, the growth of an ideal 2D (N+1)thL on NL requires that, during the edge growth, new nucleations on the growing (N+1)thL domains are forbidden. This is equivalent to the statement that every atoms/species on (N+1)L domains must travel to their edges. This criterion can be satisfied if the diffusion mean free paths λ of those atoms/species on (N+1)L are larger than the domain sizes D, that is, $\lambda > D$. Note that D is equivalent to the neighboring nucleus-nucleus distance (marked in Fig. S2b), if these nuclei are uniform in sizes and spatial distributions.

III: Optimizations of growth conditions for 2D growth in each layer

As discussed above, both nucleation and edge growth are determining kinetic processes during the layered growth of MoS_2. To achieve an ideal 2D layer-by-layer growth mode in each individual layers, the criteria are: 1) during nucleation, *only monolayer* MoS_2 *nucleus are allowed*; 2) during edge growth (till layer completion), $\lambda > D$. The former criterion can be satisfied by using a high surface energy substrate, like sapphire in the present case, to tune the surface energies of MoS_2 on its top, as suggested in Part-**I.** While the latter criterion can be satisfied by tuning the practical growth parameters, as will be discussed in details.

In this work, heteroepitaxy of 1stL on sapphire was grown via condition 1 and homoepitaxy of NthL on (N-1)L with N ≧ 2 was grown via condition 2, as described in the manuscript. The difference between two conditions is that, in condition 2, temperatures of both Mo-source (T_{Mo}) and substrate ($T_{substrate}$) are elevated. All our growth were carried out via oxygen-assisted CVD at the low pressure of ~1 Torr (Ar and O_2 gas pressure) and the high $T_{substrate}$>900 ºC by using large amount of S and tiny amount of MoO_3 (i.e., S-rich atmosphere). Note that we have optimized the growth parameters previously[7,8] for 1stL on sapphire. It is also worth mentioning that, we firstly developed this oxygen-assisted CVD method and found that it is very efficient to achieve ultra-high edge growth rate of 1L-MoS_2 on sapphire, e.g. ~5-10 μm/min, compared to that less than ~0.1 μm/min without the oxygen assistance. This enhancement is attributed to the passivation of the MoS_2 domain edges by oxygen, decreasing the reaction energy barrier during the kinetic growth process.

After the completion of 1L-MoS_2/sapphire, we then tried to continue the next layer homoepitaxy. It turns out that, after 1L completion, repeating condition 1 would yield small 2L domains rather than a complete 2L due to substrate changes, as shown in below Fig. S4a. It can also seen that the as-grown triangular domains have nearly identical sizes, suggesting that the nucleation of 2L has already reached the saturation. In order to improve the nucleation density, as well as the edge growth rate, we thus enhanced the Mo-flux by increasing T_{Mo}. A series of control experiments carried out at various T_{Mo} (while keeping other growth parameters the same) indeed show this strategy is effective, confirmed from the improved nucleation density of 2ndL on 1L by ~4 times from T_{Mo}=540 ºC to T_{Mo}=570 ºC (below Fig. S4b).

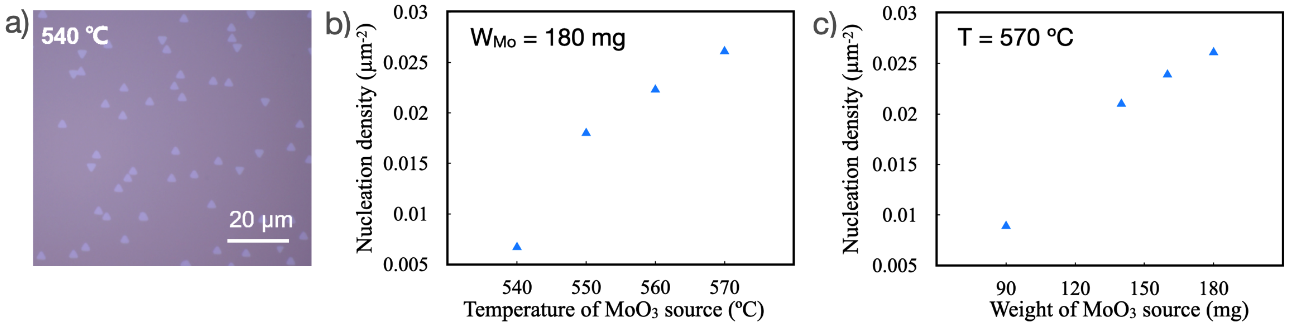

Fig. S4 Nucleations of 2L on the complete 1L. a) Optical image of a sample of 2L domains on a complete 1L grown by repeating the condition 1 for one time. b)/c) Nucleation density of 2L on a complete 1L tuned by the Mo-source temperature/weight. For samples shown in b)/c), only Mo-source temperatures/amounts were changed while the other growth parameters are the same as those in condition 1/2. From these data, we can see that the 30 ºC increment of T_{Mo} has a similar effect to the doubled weight of W_{Mo} in terms of increasing the nucleation density. Since our growth runs are continuous, we thus adopted the Mo-source temperature increase strategy in all our growth.

Experimentally, we choose T_{Mo}=570 ºC, $T_{substrate}$=940 ºC for an optimized growth (condition 2). The 30 ºC increase for $T_{substrate}$ over condition 1 is used to balance the growth time. The time evolution of growth of a complete 2L-MoS_2/sapphire is shown in the below Fig. S5). We can see that the 2D growth 1stL on sapphire is nearly perfect (Fig. S5a-b). Sizes of these triangular domains are very large, mostly over ~200 μm, but with a certain size distribution. Both domain sizes and distributions suggest that the heteroepitaxy process follows case 1 mentioned in above Part-**II**.

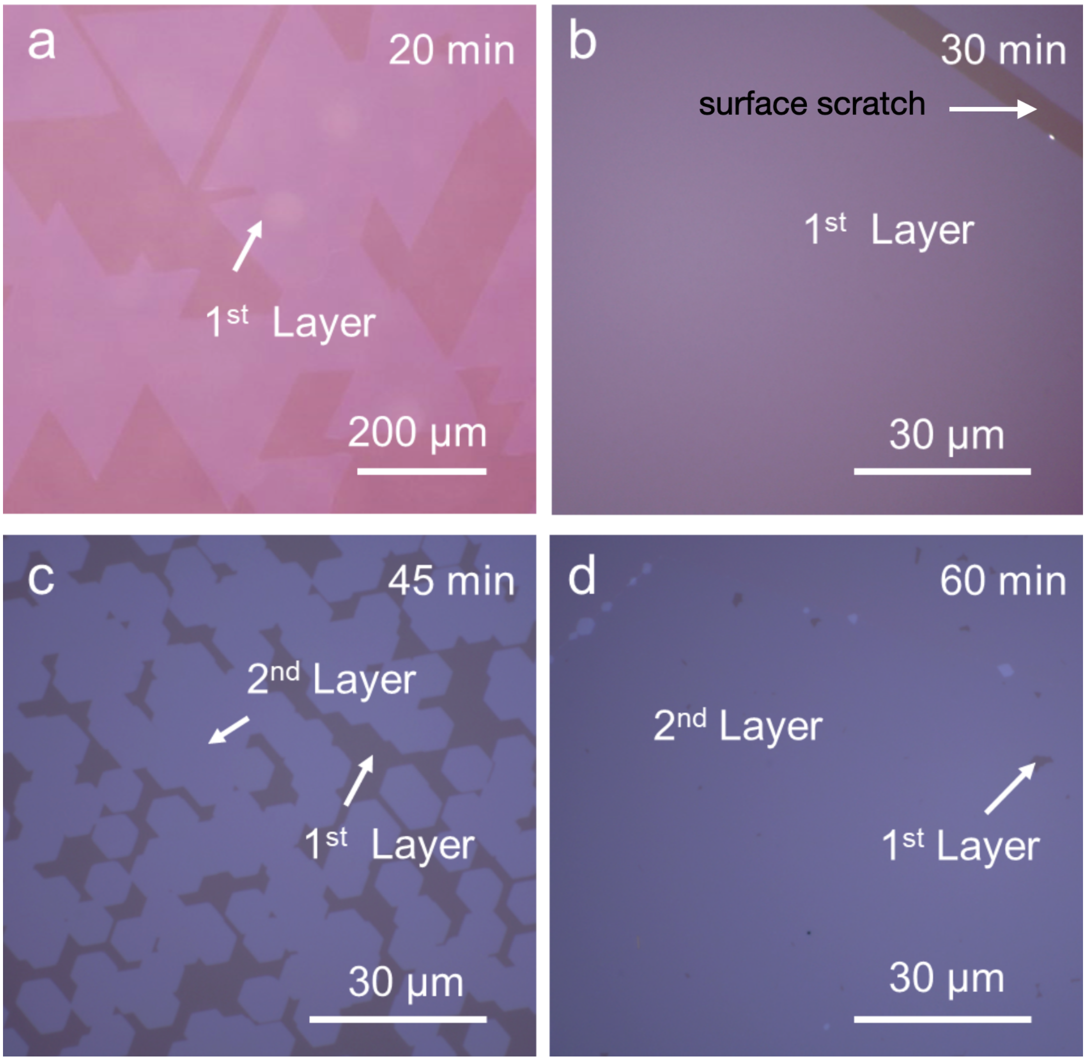

Fig. S5 Time evolution of bilayer MoS_2 continuous film growth. Optic images of 0.7L (a), 1L (b), 1.7L (c), and 2L (d) MoS_2 film. Decimals, e.g. 1.7, denotes fully covered first-layer with 70% coverage of the second-layer domains.

From Fig. S5c-d, we can also see that the 2D growth 2ndL-MoS_2 is also nearly perfect. The 2ndL nucleation distributes uniformly and sizes of these hexagonal domains are nearly identical at ~10 µm, suggesting that the homoepitaxy process follows case 2 mentioned in Part-**II**. Note that the shape of a domain is controlled by the specific edge (Mo-terminated or S-terminated) growth rate V, the hexagonal shape corresponds to $V_{Mo} \approx V_{Mo}$ (otherwise it is triangular). Here, the hexagonal rather than triangular shapes are beneficial, as they are easier to be connected and coalesced for final layer completion. Besides, it is also noticed that the edge growth rate between heteroepitaxy (V_{hetero}) and homoepitaxy (V_{homo}) is significantly different, e.g. V_{hetero} of ~7 µm/min while V_{homo} of ~0.4 µm/min. This difference is attributed to the higher adsorption or lower desorption rate of Mo-atoms/species on sapphire surface, due to the existence of many dangling bonds.

Further repeating the growth by condition 2 thus yields 3L, 4L, and so on, on sapphire. As can be seen in Fig. 1 in the main manuscript, additional small 4L domains appear on the fully covered 3L, and the 3D growth tends to be more significant for even thicker layers. Based on the above analysis, we could understand this from two aspects. Firstly, the mean free path issue should be considered. For growth condition 2, the greater the Mo-flux, the shorter the λ_{Mo} on the MoS_2 surface is. As a consequence, the nucleation density and domain sizes need to be balanced. If $\lambda > D$, it leads to the 3D growth. Secondly, the monolayer nucleations on the thicker layer (N>3) tends to fail, as suggested by above thermodynamics analysis in Part-**I**.

IV: Summary of the Layer-by-layer growth process

Based on the above analysis together with the discussions in the main manuscript, we summarize key factors to achieve 2D growth of MoS_2: 1) the surface proximity effect; 2) control of nucleation and edge growth in the kinetic growth process. The layer-by-layer growth flow chart for 1L, 2L, and 3L epitaxial growth in this work is given in below Fig. S6.

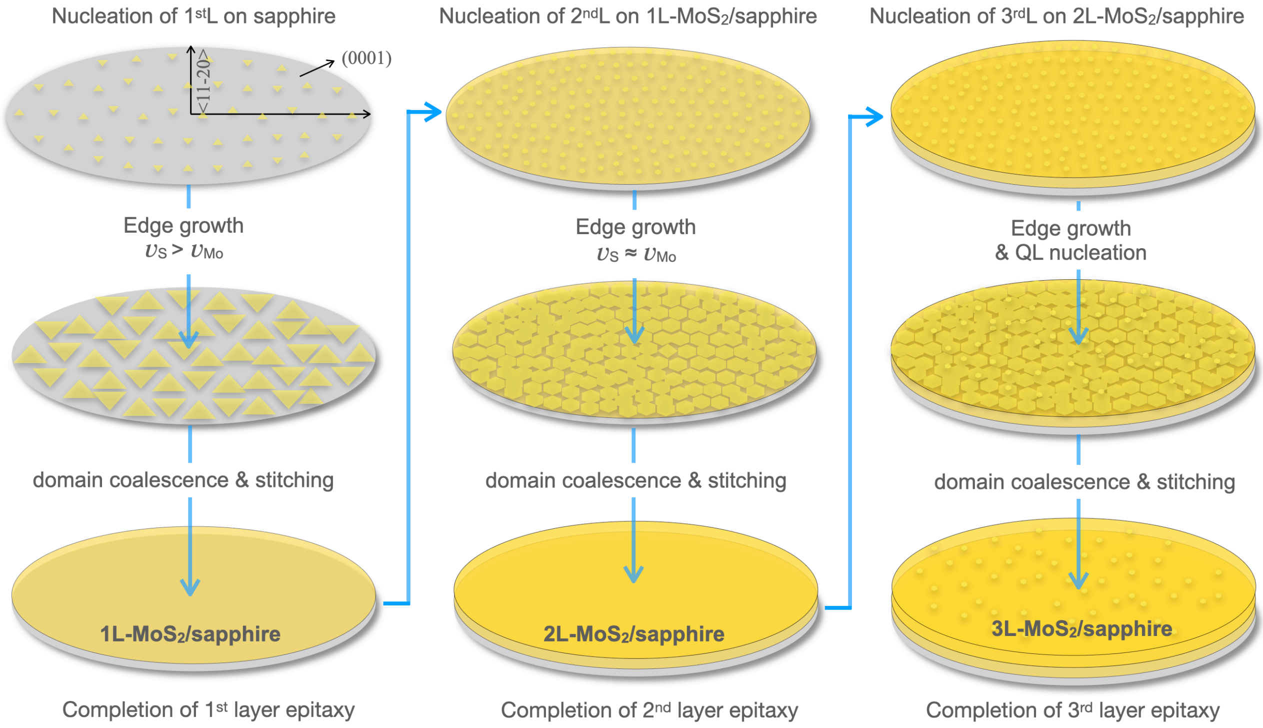

Fig. S6 Illustration of the layer-by-layer growth process for ML-, BL-, and TL-MoS_2 continuous films on a 4-inch sapphire wafer. Sapphire wafer crystallographic plane of (0001) and direction of <11-20> are marked by the black arrows.

PART 2: Additional Characterization Data and Illustrations

I: Uniformity of 2L nuclei

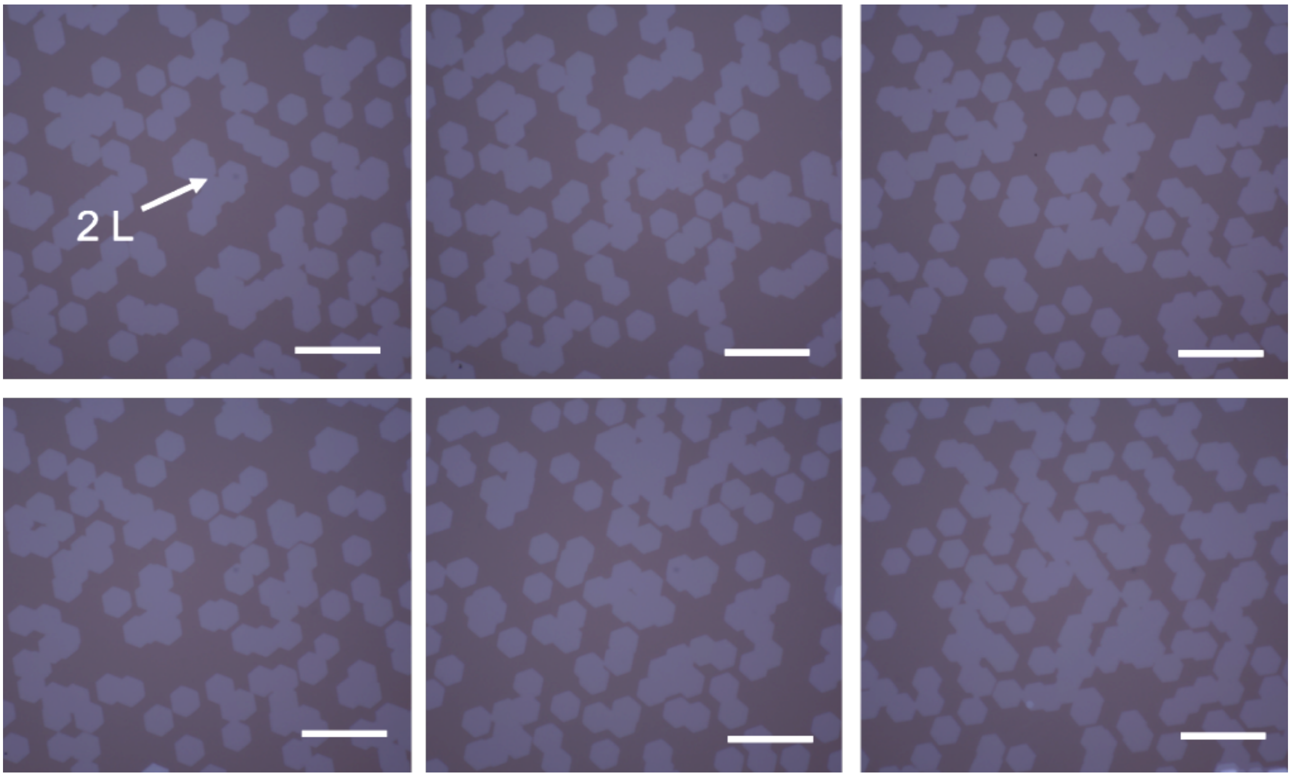

Fig. S7 Homogeneous 2L nuclei on 1L at wafer scale. Optic images of the second layer MoS_2 domains on monolayer taking from different locations across the entire 4-inch wafer. Scale bars, 20 μm.

II: Characterizations of multilayer 3L-, 4L- and 6L-MoS_2 films

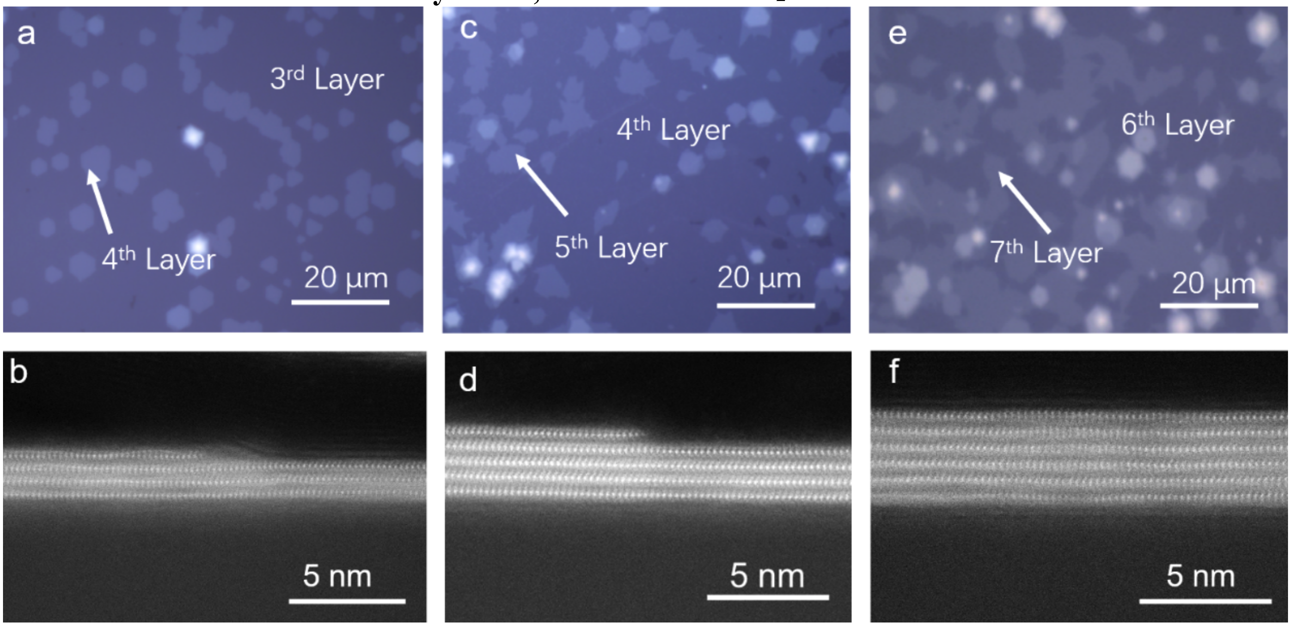

Fig. S8 Optical images and cross-sectional STEM images of multilayer MoS_2 continuous films. (a/b) 3L, (c/d) 4L, (e/f) 6L.

III: Uniformity of a continuous 3L-MoS_2 at wafer scale

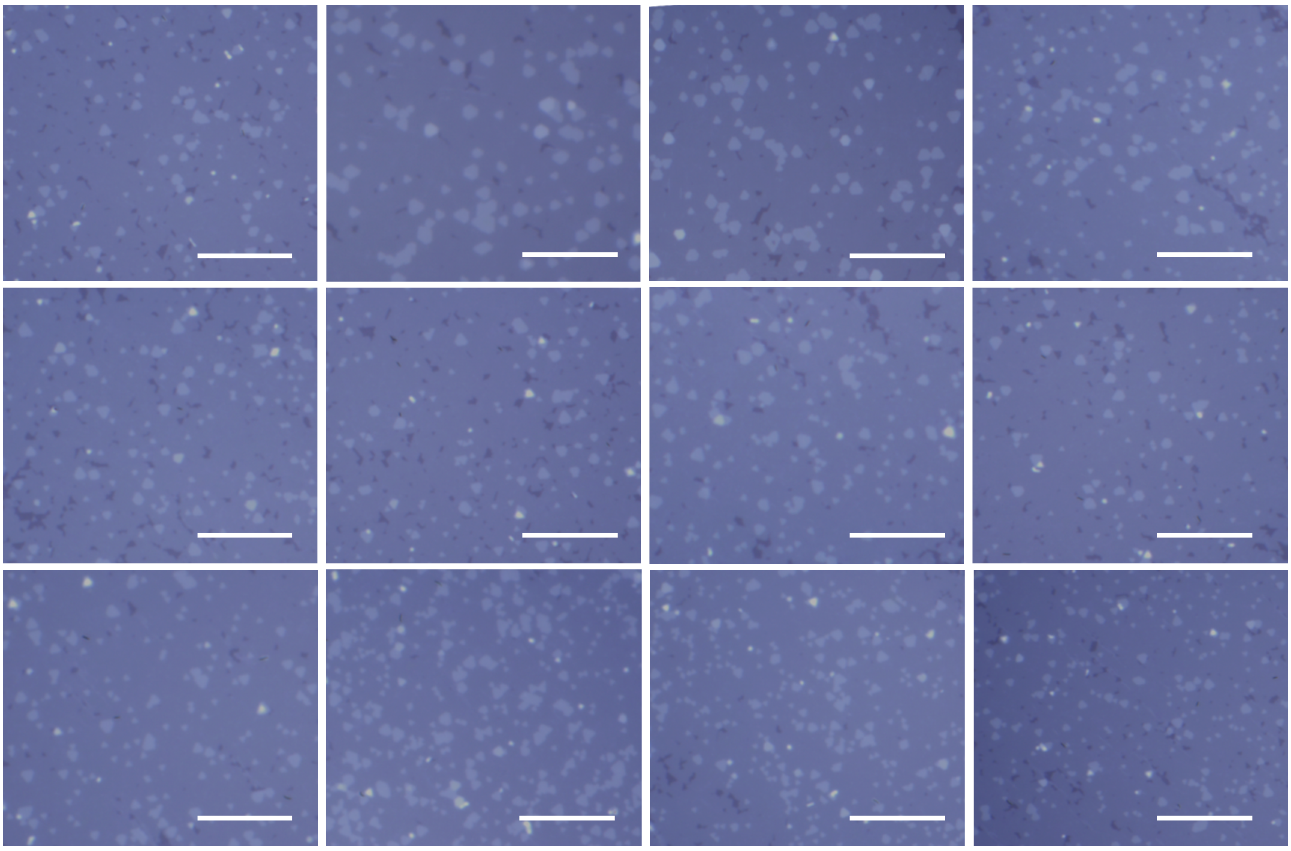

Fig. S9 Optic images of a as-grown 3L-MoS_2/sapphire samples taking from many locations across the entire 4-inch wafer. Scale bars, 30 µm.

IV: Atomic configurations of the differently stacked trilayer MoS_2

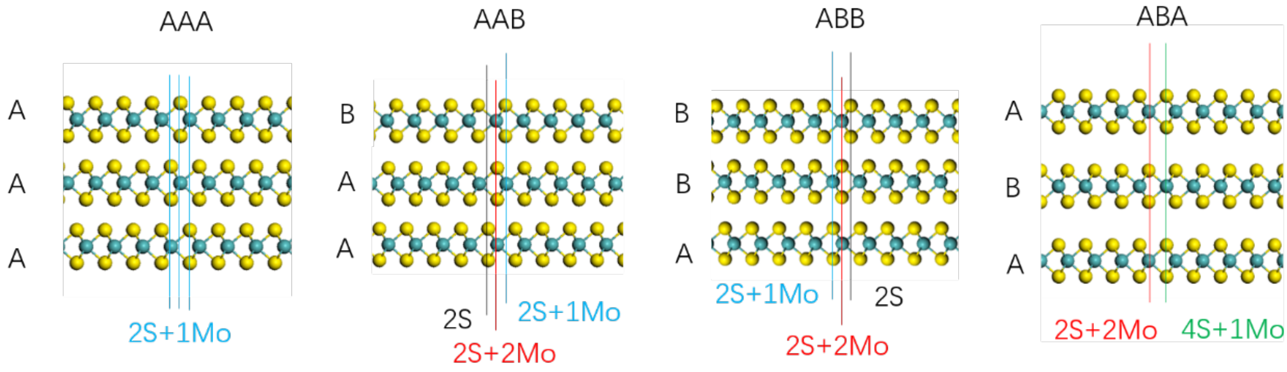

Fig. S10 Side view in ball-and-stick mode of the atomic structures of trilayer MoS_2 with different stacking orders AAA, AAB, ABB and ABA.

V: SHG mapping of bilayer MoS_2 film

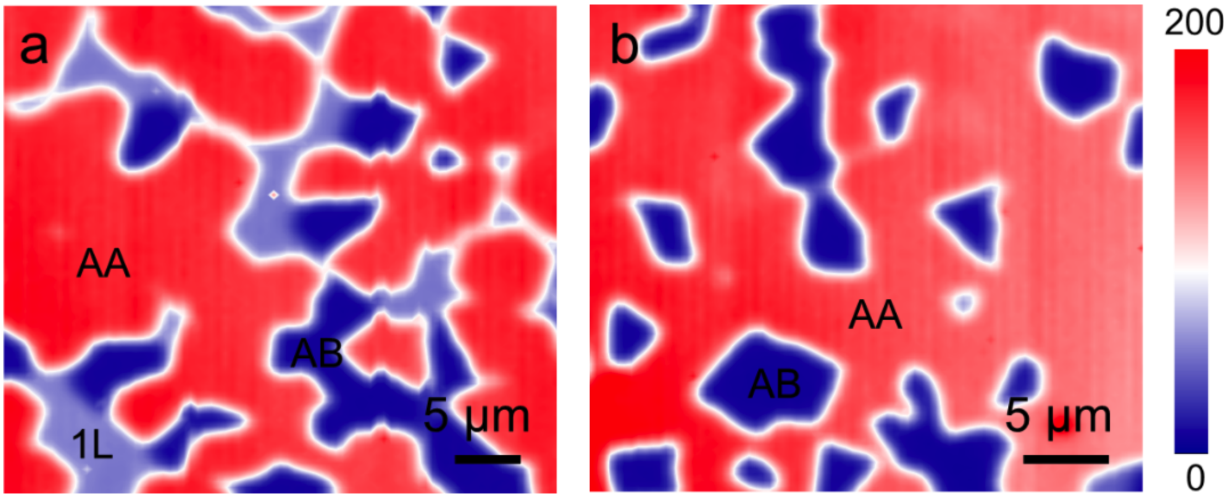

Fig. S11 (a)/(b) SHG mapping for a partially/fully completed bilayer MoS_2 film with/without discontinuous area within the 2nd layer. Note that the shape of domains in the 1st layer (triangular) and in the 2nd layer (hexagonal) are no longer recognized by SHG mapping due to the merging of domains.

VI: Working flow of the device fabrication process

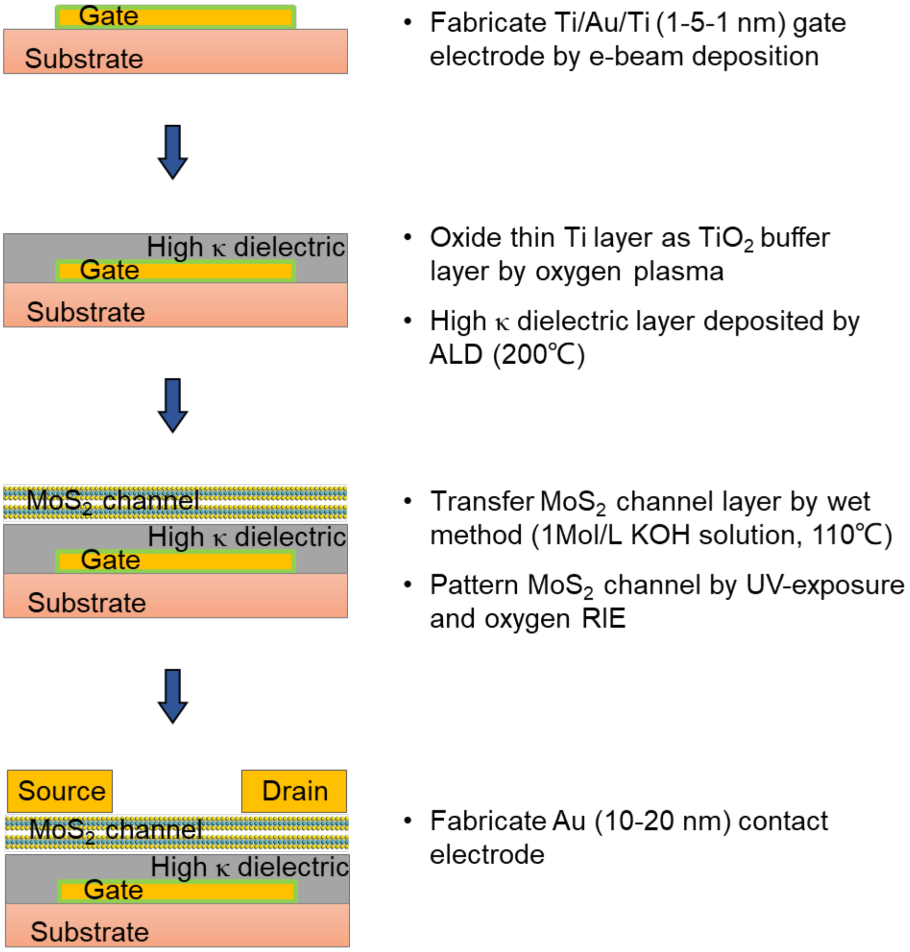

Fig. S12 Schematic illustration of the back-gated MoS_2 FET fabrication process.

VII: Low-bias performance of the 40-nm short channel device

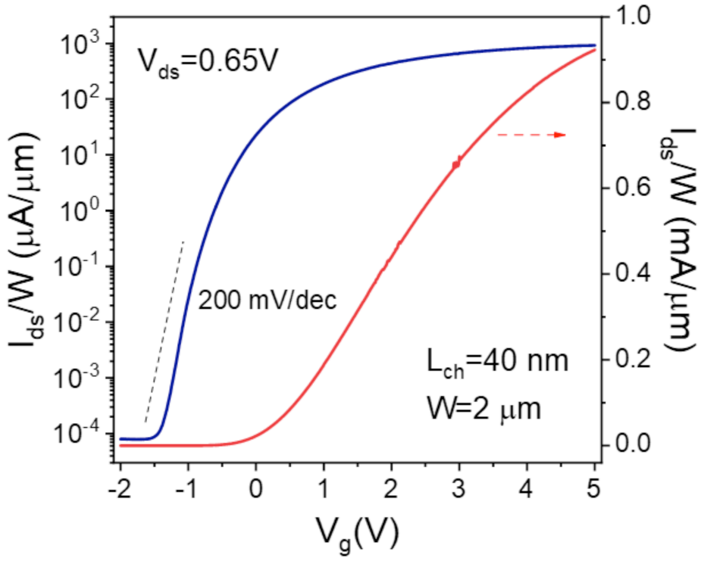

Fig. S13 Transfer curves of the 40-nm channel length device measure at V_{ds}=0.65 V.

VIII: Comparison of 1L-, 2L- and 3L-MoS_2 short channel devices

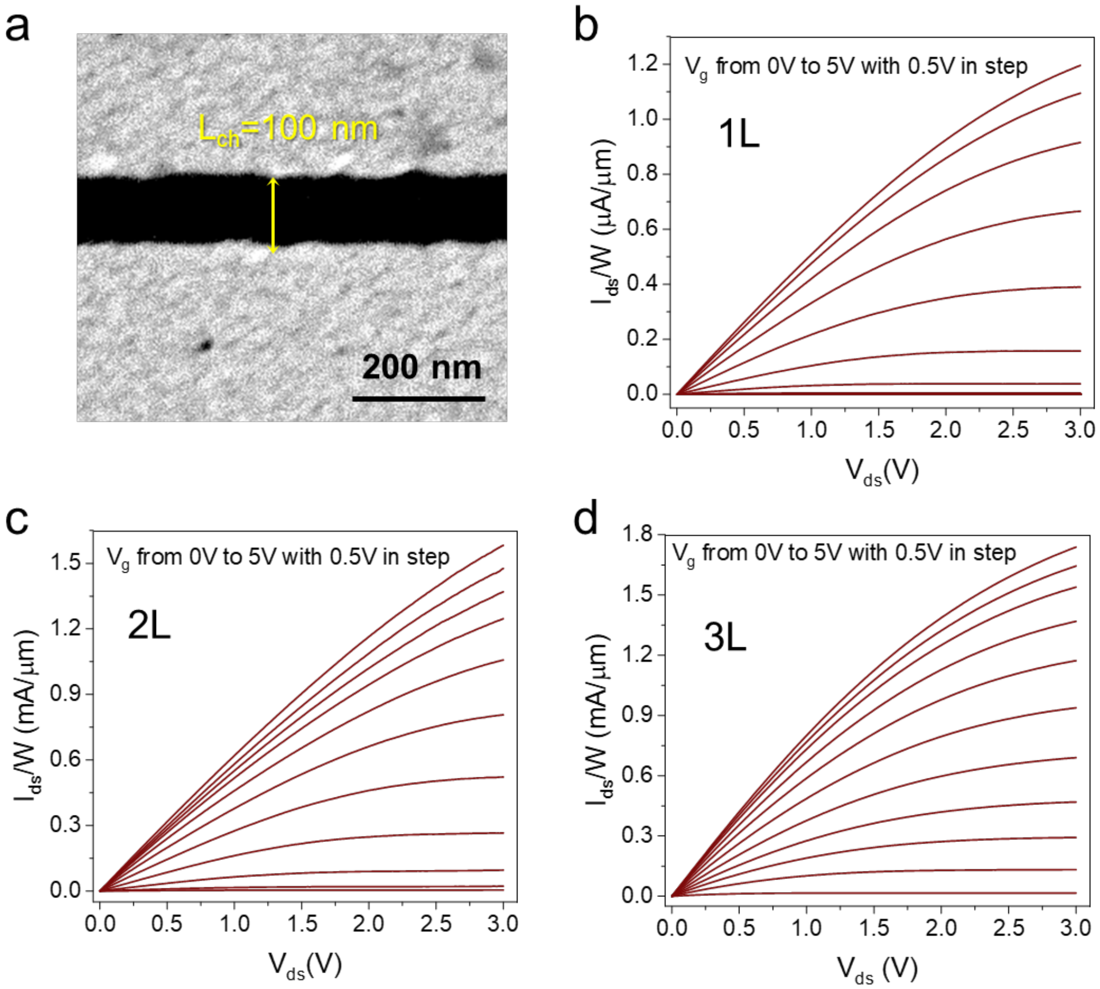

Fig. S14 Output curves of monolayer, bilayer and trilayer MoS_2 FETs at L_{ch}=100 nm. (a) SEM image of MoS_2 FET with L_{ch}=100 nm. (b) The output curves of monolayer MoS_2 FET. (c) The output curves of bilayer MoS_2 FET. (d) The output curves of trilayer MoS_2 FET.

IX: Transfer curves of 1L- and 2L-MoS_2 FETs

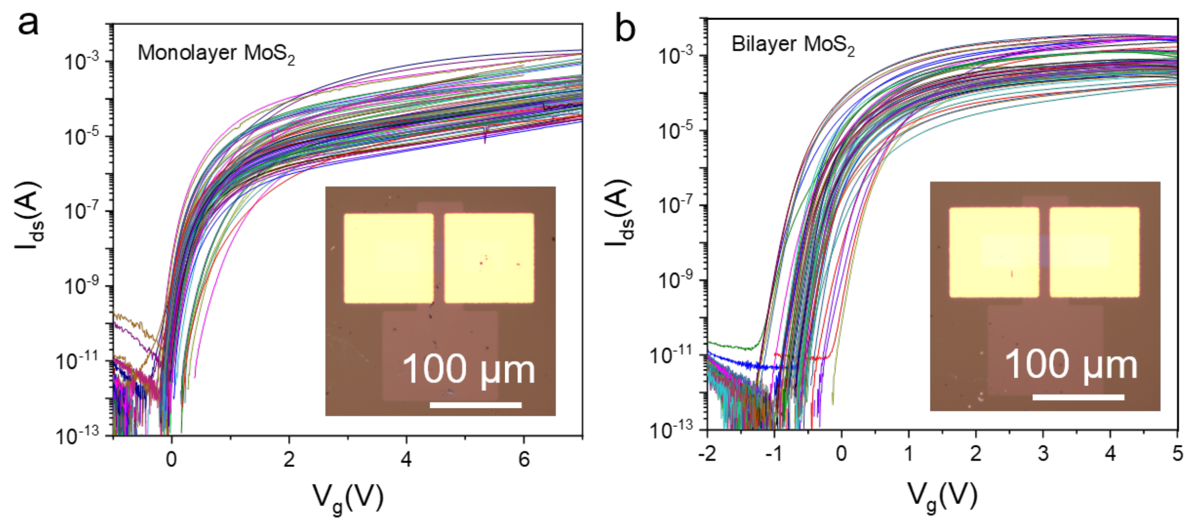

Fig. S15 Transfer curves of 150 randomly picked 1L/2L MoS_2 FETs. Inset is the optic image of a typical device. Note that these 1L/2L MoS_2 FETs have channel length L_{ch} varying from 5 μm to 50 μm and channel width W_{ch} varying from 10 μm to 30 μm.

X: Transfer curves of 3L-MoS_2 FETs

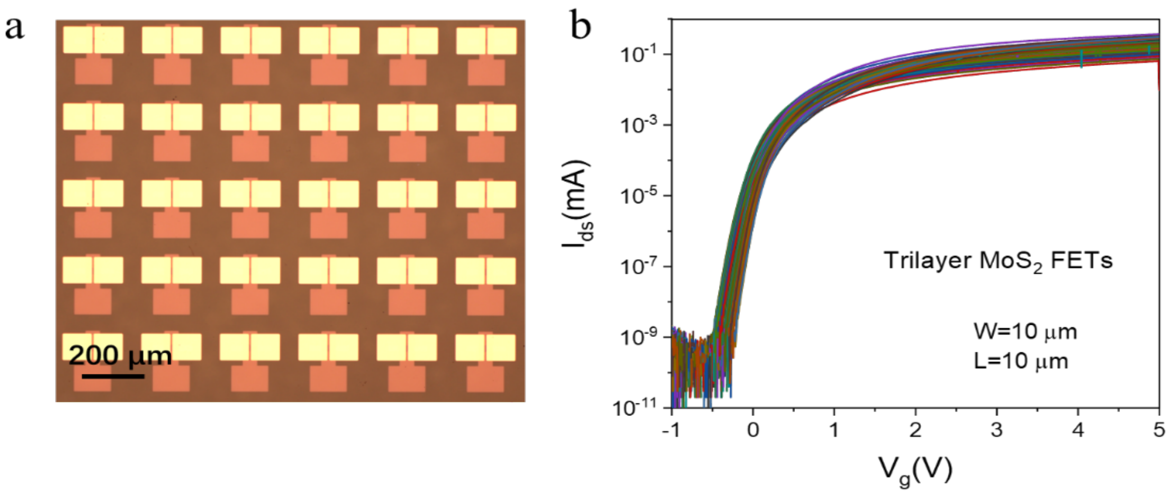

Fig. S16 Transfer curves of 100 randomly picked 3L-MoS_2 FETs with same L_{ch}=10 μm and W_{ch}=10 μm. The zoom-in optic image of devices array is shown in (a).

XI: Comparison of different approaches to produce MoS_2 multilayers

Table 1. Multilayer flakes

Method	Number of layers	Flake size	Electron Mobility	On/Off ratio	References
Sulfurization	1L - 4 L	10 µm	0.1-0.7	10^6	Ref. 9
CVD	1L - N L	~50 µm	20-67	10^6	Ref. 10
CVD	2L	~200 µm	36	10^7	Ref. 11
CVD	2L	~20 µm	21	10^7	Ref. 12
CVD	1L - 5L	~100 µm	/	10^7	Ref. 13
CVD	2L	100-300 µm	7-75	10^4	Ref. 14
CVD	2L	20-30 µm	32.6	10^7	Ref. 15
CVD	2L	(Nanoribbons)	4	10^4	Ref. 16

Table 2. Multilayer films

Method	Film thickness	Film size	Domain alignment	Domain size	Electron Mobility	On/Off ratio	References
Sulfurization	1.8-2.0 nm	2 inch	random	/	~0.8	10^5	Ref. 17
Sulfurization	1L - fewL	~ 1cm	random	<100 nm	<0.04	/	Ref. 18
Sulfurization	~2 nm	~5 cm	random	/	<10	10^5	Ref. 19
Sulfurization	3-4 nm	4 inch	random	5-7 nm	~3.74	/	Ref. 20
CVD on SiO_2	1L/2L/3 L	~2 cm	random	/	3.6/8.2/15.6	10^6	Ref. 21
ALD on SiO_2 or sapphire	~3 nm	2 inch	random	/	~0.56	10^6	Ref. 22

CVD on sapphire (LBL epitaxy)	1L/2L/3L	4 inch	aligned	~200/10/10 μm @ 1L/2L/3L	~80/110/145 @ 1L/2L/3L	10^8-10^9	This work

XII: Comparison of RT performances of various MoS_2 FETs

Table S3:

Layer number	Channel length (L_{ch})	On/off ratio	I_{ds}/W @ V_{ds}=1V (μA/μm)	I_{on} max (μA/μm)/ V_{ds} (V)	Mobility ($cm^2V^{-1}s^{-1}$)	References
1L	35 nm	10^6	840	1135/1.5	22	Ref. 23
1L	82 nm	$4*10^3$	466	/	23.2	Ref. 24
1L	112 nm	$2*10^6$	229	303/1.4	8.1	Ref. 24
1L	380 nm	10^6	280	700/5	33.5	Ref. 25
1L	10 nm	$5*10^7$	190	540/3.5	30	Ref. 26
6L	100 nm	$6.3*10^5$	350	460/1.6	50	Ref. 27
4L	35 nm	10^8	250	/	4	Ref. 28
1L	50 nm	10^8	156	250/1.6	20	Ref. 29
6L	80 nm	$6*10^5$	640	830/2	51	Ref. 30
5L	100 nm	10^7	350	510/3	/	Ref. 31
8L	100 nm	10^8	45	87/2	20	Ref. 32
2L	250 nm	10^7	12	28/2.5	/	Ref. 33
1L	500 nm	10^9	140	450/6	77.6	Ref. 34
2L	1000 nm	10^7	23	/	15	Ref. 35
3L	1200 nm	10^8	85	/	46	Ref. 36
1L	1900 nm	10^{10}	11	44/4	/	Ref. 37
16L	5000 nm	10^7	60	240/5	/	Ref. 38
1L	6000 nm	10^{10}	13	35/3	55	Ref. 39
2L	40 nm	10^6	300	427/2	30	Ref. 40
1L	2000 nm	$2*10^6$	18	/	167	Ref. 41
1L	100 nm	$7.2*10^8$	402.17	1195.5/3	80	This work

2L	100 nm	$2.27*10^8$	641.67	1579.8/3	110	This work
3L	100 nm	$6.76*10^7$	804.7	1738.7/3	145	This work
3L	40 nm	$1.5*10^7$	1221.9	1704.6/2	145	This work

PART 3: References

1. Bauer, E., & van der Merwe, J. H. Structure and growth of crystalline superlattices: From monolayer to superlattice. *Phys. Rev. B* **33**, 3657, (1986).
2. Shang, S. L., Lindwall, G., Wang, Y., Redwing, J. M., Anderson, T., & Liu, Z. K. Lateral versus vertical growth of two-dimensional layered transition-metal dichalcogenides: thermodynamic insight into MoS_2. *Nano letter.* **16**, 5742-5750, (2016).
3. Bakholdin, S. I., & Maslov, V. N. Simulation of surface energies of sapphire crystals. *Physics of the Solid State* **57**, 1236-1243, (2015).
4. Kresse, G. & Furthmüller, J. Efficient iterative schemes for ab initio total-energy calculations using a plane-wave basis set. *Phys. Rev. B* **54**, 11169, (1996).
5. Blöchl, P. E. Projector augmented-wave method. *Phys. Rev. B* **50**, 17953, (1994).
6. Perdew, J. P., Burke, K. & Ernzerhof, M. Generalized gradient approximation made simple. *Phys. Rev. Lett.* **77**, 3865, (1996).
7. Yu, H., Liao, M., Zhao, W., Liu, G., Zhou, X. J., Wei, Z., et. al., Wafer-scale growth and transfer of highly-oriented monolayer MoS_2 continuous films. *ACS nano* **11**, 12001-12007, (2017).
8. Wang, Q., Li, N., Tang, J., Zhu, J., Zhang, Q., Jia, Q., et. al., Wafer-scale highly oriented monolayer MoS_2 with large domain sizes. *Nano Letter.* ***20***, 7193-7199, (2020).
9. Wang, X., Feng, H., Wu, Y., & Jiao, L. Controlled synthesis of highly crystalline MoS_2 flakes by chemical vapor deposition. *J. Am. Chem. Soc.* **135**, 5304-5307, (2013).
10. Zheng, J., Yan, X., Lu, Z., Qiu, H., Xu, G., Zhou, X., et. al, High-mobility multilayered MoS_2 flakes with low contact resistance grown by chemical vapor deposition. *Adv. Mater.* **29**, 1604540, (2017).
11. Gao, Q., Zhang, Z., Xu, X., Song, J., Li, X., & Wu, Y. Scalable high performance radio frequency electronics based on large domain bilayer MoS_2. *Nat. Commun.* **9**, 1-8, (2018).
12. Fang, M., Wang, F., Han, Y., Feng, Y., Ren, T., Li, Y., et. al, Controlled growth of bilayer-MoS_2 films and MoS_2-based field-effect transistor (FET) performance optimization. *Adv. Electron. Mater.,* **4**, 1700524, (2018).
13. Yang, P., Zhang, Z., Sun, M., Lin, F., Cheng, T., Shi, J., et. al, Thickness tunable wedding-cake-like MoS_2 flakes for high-performance optoelectronics. *ACS nano*, **13**, 3649-3658, (2019).
14. Zhang, X., Nan, H., Xiao, S., Wan, X., Gu, X., Du, A., et. al, Transition metal dichalcogenides bilayer single crystals by reverse-flow chemical vapor epitaxy. *Nat. Commun.* **10**, 1-10, (2019).
15. Li, B., Ju, Q., Hong, W., Cai, Q., Lin, J., & Liu, W. Edge defect-assisted synthesis of chemical vapor deposited bilayer molybdenum disulfide. *Ceramics International* **47**, 30106-30112, (2021).
16. Li, X., Li, B., Lei, J., Bets, K. V., Sang, X., Okogbue, E., et. al, Nickel particle–enabled width-controlled growth of bilayer molybdenum disulfide nanoribbons. *Science Advances* **7**, eabk1892, (2021).
17. Lin, Y. C., Zhang, W., Huang, J. K., Liu, K. K., Lee, Y. H., Liang, C. T., et. al, Wafer-scale MoS_2 thin layers prepared by MoO_3 sulfurization. *Nanoscale* **4**, 6637-6641. (2012).
18. Zhan, Y., Liu, Z., Najmaei, S., Ajayan, P. M., & Lou, J. Large-area vapor-phase growth and characterization of MoS_2 atomic layers on a SiO_2 substrate. *Small* **8**, 966-971, (2012).
19. Liu, K. K., Zhang, W., Lee, Y. H., Lin, Y. C., Chang, M. T., Su, C. Y., et. al, Growth of large-area and highly crystalline MoS_2 thin layers on insulating substrates. *Nano letter.* **12**, 1538-1544, (2012).
20. Ahn, C., Lee, J., Kim, H. U., Bark, H., Jeon, M., Ryu, G. H., et. al, Low-temperature synthesis

of large-scale molybdenum disulfide thin films directly on a plastic substrate using plasma-enhanced chemical vapor deposition. *Adv. Mater.* **27**, 5223-5229, (2015).

21. Jeon, J., Jang, S. K., Jeon, S. M., Yoo, G., Jang, Y. H., Park, J. H., & Lee, S. Layer-controlled CVD growth of large-area two-dimensional MoS_2 films. *Nanoscale* **7**, 1688-1695, (2015).
22. Liu, H., Chen, L., Zhu, H., Sun, Q. Q., Ding, S. J., Zhou, P., & Zhang, D. W. Atomic layer deposited 2D MoS_2 atomic crystals: From material to circuit. *Nano Research* **13**, 1644-1650, (2020).
23. Shen, P.-C. *et al.* Ultralow contact resistance between semimetal and monolayer semiconductors. *Nature* **593**, 211-217, (2021).
24. Daus, A. *et al.* High-performance flexible nanoscale transistors based on transition metal dichalcogenides. *Nat. Electron.* **4**, 495-501, (2021).
25. McClellan, C. J., Yalon, E., Smithe, K. K. H., Suryavanshi, S. V. & Pop, E. High Current Density in Monolayer MoS_2 Doped by AlO_x. *ACS Nano* **15**, 1587-1596, (2021).
26. Xie, L. *et al.* Graphene-Contacted Ultrashort Channel Monolayer MoS_2 Transistors. *Adv. Mater.* **29**, 1702522, (2017).
27. Yang;, L. et al. High-Performance MoS_2 Field-Effect Transistors Enabled by Chloride Doping Record low contact resistance (0.5khom) and record high drain current 460 $\mu A \cdot \mu m^{-1}$. *2014 Symposium on VLSI Technology (VLSI-Technology): Digest of Technical Papers*, 1-2, (2014).
28. Smets, Q. et al. Ultra-scaled MOCVD MoS_2 MOSFETs with 42 nm contact pitch and 250 µA/um drain current. *2019 IEEE International Electron Devices Meeting (IEDM)*, 23.2.1-23.2.4, (2019).
29. Zhu, Y. *et al.* Monolayer Molybdenum Disulfide Transistors with Single-Atom-Thick Gates. *Nano Lett.* **18**, 3807-3813, (2018).
30. Liu, Y. *et al.* Pushing the Performance Limit of Sub-100 nm Molybdenum Disulfide Transistors. *Nano Lett.* **16**, 6337-6342, (2016).
31. Si, M. *et al.* Steep-slope hysteresis-free negative capacitance MoS_2 transistors. *Nat. Nanotechnol.* **13**, 24-28, (2018).
32. Liu, H., Neal, A. T. & Ye, P. D. Channel length scaling of MoS_2 MOSFETs. *ACS Nano* **6**, 8563-8569, (2012).
33. Desai, S. B. *et al.* MoS_2 transistors with 1-nanometer gate lengths. *Science* **354**, 99-102, (2016).
34. 34. Li, T. *et al.* Epitaxial growth of wafer-scale molybdenum disulfide semiconductor single crystals on sapphire. *Nat. Nanotechnol.* (2021). https://doi.org/10.1038/s41565-021-00963-8.
35. Wang, H. *et al.* Integrated circuits based on bilayer MoS_2 transistors. *Nano Lett.* **12**, 4674-4680, (2012).
36. Kappera, R. *et al.* Phase-engineered low-resistance contacts for ultrathin MoS_2 transistors. *Nat. Mater.* **13**, 1128-1134, (2014).
37. Zheng, X. *et al.* Patterning metal contacts on monolayer MoS_2 with vanishing Schottky barriers using thermal nanolithography. *Nat. Electron.* **2**, 17-25, (2019).
38. Das, S., Chen, H. Y., Penumatcha, A. V. & Appenzeller, J. High performance multilayer MoS_2 transistors with scandium contacts. *Nano Lett.* **13**, 100-105, (2013).
39. Li, N. *et al.* Large-scale flexible and transparent electronics based on monolayer molybdenum disulfide field-effect transistors. *Nat. Electron.* **3**, 711-717, (2020).
40. Gao, Q. *et al.* Scalable high performance radio frequency electronics based on large domain bilayer MoS_2. *Nat. Commun.* **9**, 4778, (2018).
41. Wang, Y. *et al.* Van der Waals contacts between three-dimensional metals and two-dimensional semiconductors. *Nature* **568**, 70-74, (2019).
